# *In silico* analyses identify lncRNAs: WDFY3-AS2, BDNF-AS and AFAP1-AS1 as potential prognostic factors for patients with triple-negative breast tumors

**DOI:** 10.1371/journal.pone.0232284

**Published:** 2020-05-13

**Authors:** Daniel Rodrigues de Bastos, Maria A. Nagai

**Affiliations:** 1 Discipline of Oncology, Department of Radiology and Oncology, Faculty of Medicine, University of São Paulo, São Paulo, Brazil; 2 Laboratory of Molecular Genetics, Center for Translational Research in Oncology, Cancer Institute of São Paulo, São Paulo, Brazil; University of Kansas Medical Center and VA Medical Center, UNITED STATES

## Abstract

**Background:**

Long non-coding RNAs (lncRNAs) are characterized as having 200 nucleotides or more and not coding any protein, and several been identified as differentially expressed in several human malignancies, including breast cancer.

**Methods:**

Here, we evaluated lncRNAs differentially expressed in triple-negative breast cancer (TNBC) from a cDNA microarray data set obtained in a previous study from our group. Using *in silico* analyses in combination with a review of the current literature, we identify three lncRNAs as potential prognostic factors for TNBC patients.

**Results:**

We found that the expression of WDFY3-AS2, BDNF-AS, and AFAP1-AS1 was associated with poor survival in patients with TNBCs. WDFY3-AS2 and BDNF-AS are lncRNAs known to play an important role in tumor suppression of different types of cancer, while AFAP1-AS1 exerts oncogenic activity.

**Conclusion:**

Our findings provided evidence that WDFY3-AS2, BDNF-AS, and AFAP1-AS1 may be potential prognostic factors in TNBC development.

## Introduction

Breast cancer represents the second most common type of cancer worldwide and with high morbidity and mortality rates considering only females [1]. Breast cancer shows high heterogeneity, which impacts the clinical course of the disease [2]. Differences in the expression profile among patients contribute to the heterogeneity that gives the tumor tissue differences in malignant behavior and impact on the prognosis and response to usual treatments [3]. Based on the expression of molecular biomarkers, breast cancer is classified into four main subtypes: Luminal A, Luminal B, HER2-positive, and triple-negative (TNBC). The Luminal A and B breast cancer subtypes express hormone receptors, estrogen receptor (ER) and progesterone receptor (PR). The HER2-positive subtype is negative for the hormonal receptors and shows high expression levels of the human epidermal growth factor receptor 2. The triple-negative subtype (TNBC) consists of breast tumors negative for the expression of hormone receptors and the HER2 oncoprotein [4–6].

TNBC can be classified into at least six distinct subtypes with differences in clinical behavior and treatment response [7, 8]. TNBC subtype comprises between 15 and 20% of all breast tumors and is generally considered to have a poorer prognosis as patients do not respond to endocrine therapy and target-directed therapies [9, 10]. TNBCs are commonly observed in younger and obese women, being more prevalent in premenopausal African American women [11]. Germline mutations of BRCA1 and BRCA2 are found in about 20% of patients with TNBC [12]. Also, tumors of the TNBC subtype show a high frequency of P53 and Rb1 mutations [13].

ncRNAs comprise a broad class of RNAs that can be divided into two subclasses based on their size: long non-coding RNAs and small non-coding RNAs [14]. Long non-coding RNAs (lncRNAs) are characterized by having 200 nucleotides or more and not coding for any protein [15]. lncRNAs have been identified as differentially expressed in several human malignancies [16–18], including breast cancer [19, 20]. There are several mechanisms of action attributed to these lncRNA molecules, highlighting their participation in protein recruitment, scaffold, endogenous competition for microRNAs (ceRNA) and enhancer of other genes [21, 22]. LncRNAs play an important role in cellular homeostasis, including participation in multiple pathological processes and tumorigenesis of various tissues [23, 24]. However, to date, the function of most lncRNAs has not been thoroughly characterized [25], requiring *in silico* and experimental studies to further investigate their role in cell and tumor biology.

Many lncRNA molecules have been associated with the tumorigenic process, and their differential expression may represent a promising category of potential new biomarkers. In cancer cells, the lncRNA HOTAIR (HOX transcript antisense intergenic RNA), was associated with the polycomb repressing complex 2 (PRC2) and the histone demethylation enzyme lysine-specific demethylase 1 (LSD1) resulting in epigenetic regulations that lead to the tumor growth and metastasis [26]. The overexpression of HOTAIR was observed in TNBC patients and was associated with increased cell proliferation [27] and worst prognosis [28, 29]. Besides, HOTAIR has been detected in the blood of patients, and high circulating levels were correlated with worse prognosis and less response to neoadjuvant chemotherapy [30]. Furthermore, other circulating lncRNAs, such as MALAT1, GAS5, H19, and MEG3, has been associated with survival and chemotherapy response [31–34]. LncRNAs have opened a new field of study for researchers around the world, and significant functions are attributed to these molecules, which may directly impact patient survival and therapy response [35]. In this sense, the identification of differentially expressed transcripts of lncRNAs in tumor tissues is necessary to detect new potential prognostic and predictive biomarkers in breast cancer.

The SPARC gene (secreted protein acidic and rich in cysteine, also named as osteonectin or basement-membrane protein 40) encodes for a 32 kDa matricellular glycoprotein, that has been involved in several biological processes, such as differentiation, proliferation, migration, and adhesion [36, 37]. Moreover, abnormal SPARC expression has been associated with tumor characteristics such as growth and metastasis in different cancer types [38]. Previous studies from our group evaluating the gene expression profile in breast epithelial cells with the difference in ERBB2 expression before and after treatment with Docetaxel identified differential expression of SPARC as a potential biomarker of chemosensitivity [39, 40]. Subsequently, we investigate the potential prognostic value of the SPARC protein using immunohistochemical analysis on tissue-microarrays. We found that low SPARC expression is associated with worse prognosis and more aggressive phenotypes of breast cancer, including TNBC [41]. More recently, we performed a study using cDNA microarrays to determine the expression profiling of a small subset of triple-negative breast tumors with differences in SPARC expression and clinical outcome, which lead us to identify several differentially expressed genes as potential new biomarkers candidate for TNBC [42].

In the present work, we sought to identify differentially expressed lncRNAs in TNBC with SPARC expression using a cDNA microarray data set obtained in a previous study from our group [42]. Using *in silico* analyses in combination with a review of the current literature, we examined the transcriptional profiles of lncRNAs between breast tumors and normal tissues and correlate gene expression levels to clinicopathological features and patient’s clinical outcome.

## Material and methods

### Data selection

**Fig 1:**
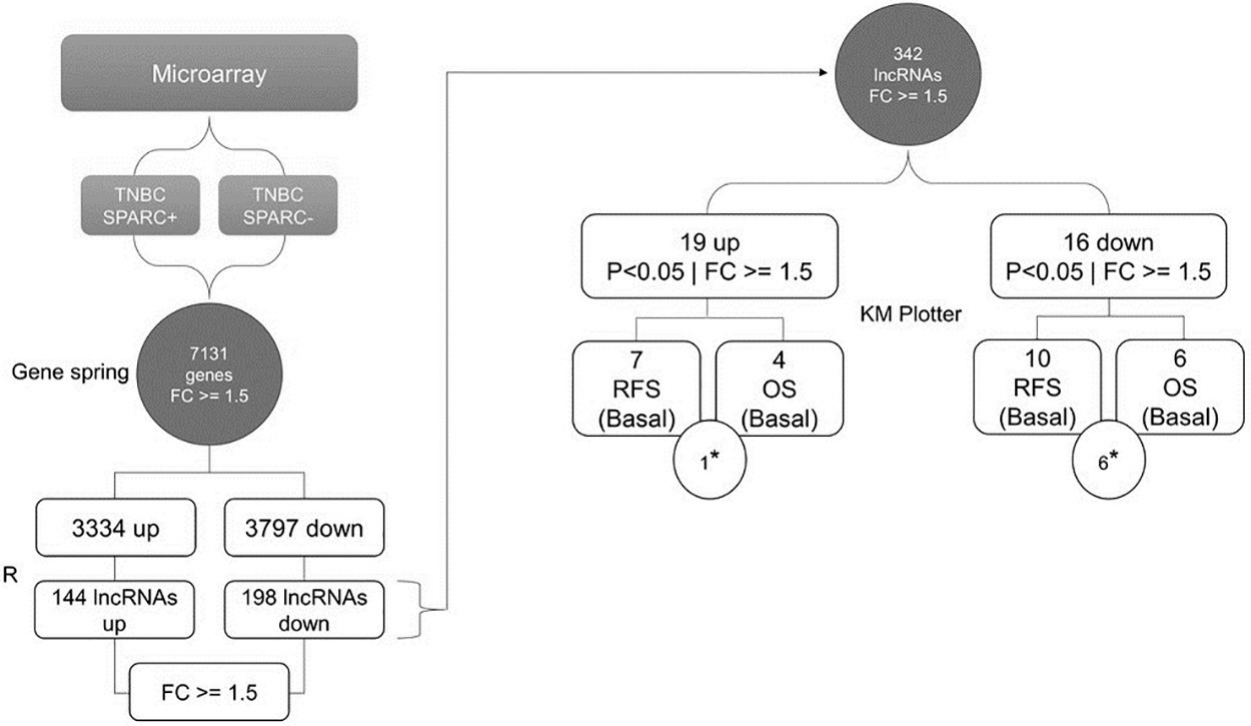
Flowchart summarizing the data selection and management for discovery of lncRNAs differentially expressed in TNBC. Selection and management of data from the microarray GSE98931. The data were initially processed in the GeneSpring software, resulting in two lists (up and down) containing coding and non-coding transcripts with a > = 1.5-fold change and a corrected p-value less than 0.05 were accepted as differentially expressed. The list was cleaned using R commands to exclude coding transcripts. The differentially expressed lncRNAs were evaluated using the KM Plotter online platform to investigate potential prognostic predictors associated with the basal subtype. We found 17 lncRNAs associated with worse RFS and 10 lncRNAs associated with low OS. *Number of common transcripts in both survival analyses.

A microarray data set, available on the Gene Expression Omnibus (GEO) online platform with access code GSE98931 [42] was reanalyzed by GeneSpring GX software (Agilent Technologies, Santa Clara, California, EUA) for identification of differentially expressed lncRNAs in SPARC positive vs. SPARC negative (fold change > = 1.5, p <0.05) after normalization and correction by Benjamini and Hochberg (Fig 1).

### Survival analyses

We analyzed the differentially expressed long noncoding RNAs and their association with overall survival (OS) and relapse free survival (RFS) in the KM Plotter online database (http://kmplot.com/analysis/) [43]. Validated probes were chosen according to the automatic best cut off selection criteria. For the survival curves, patients were stratified by the lncRNAs expression according to intrinsic subtype: all, basal, luminal A, luminal B and HER2 + [43].

### TANRIC-TCGA data

Gene expression data of 942 breast cancer patients were downloaded using the TANRIC platform (https://www.tanric.org/) [44], of which 105 consisted of adjacent tissue samples and 837 were from tumor tissue. These data were cross-referenced with information available from TCGA (https://www.cancer.gov/about-nci/organization/ccg/research/structural-genomics/tcga) and UCSC Xena (xena.ucsc.) [45] to obtain clinicopathological data such as age, status survival, tumor stage, hormone receptors and human epidermal growth factor receptor type 2 (HER2). Cases of breast cancer or adjacent male breast tissue were excluded from the study [44].

### cBioPortal data

Z-score expression data from 1.108 patients containing information about messenger RNAs (mRNAs) were downloaded on cBioPortal database (https://cbioportal.org). This information was cross-referenced with sample IDs from the TANRIC platform to pair samples for correlation analysis [46, 47].

### Expression profile and gene networks

Where applicable, we investigate the expression profile through the online UALCAN database (ualcan.path.uab.edu) [48]. Correlation analysis was conducted based on information from lncRNAs and mRNAs according to literature notes, and mRNAs found were processed in the String database (string-db.org/) to identify biological networks [49].

### Statistical analysis

The statistical analysis was performed using SPSS (Statistical Package for Social Sciences) version 25 and GraphPad Prism v. 7 (California, USA). The results considered statistically significant were that the p-value was less than 0.05 or according to the p-adjustment when appropriate. We used different statistical tests to evaluate normal distribution, association, correlation, and accomplish group comparisons, which includes Kolmogorov-Smirnov test, chi-square test or Fisher's exact test, correlation test, Mann-Whitney and Kruskal-Wallis, respectively.

## Results

**Fig 2:**
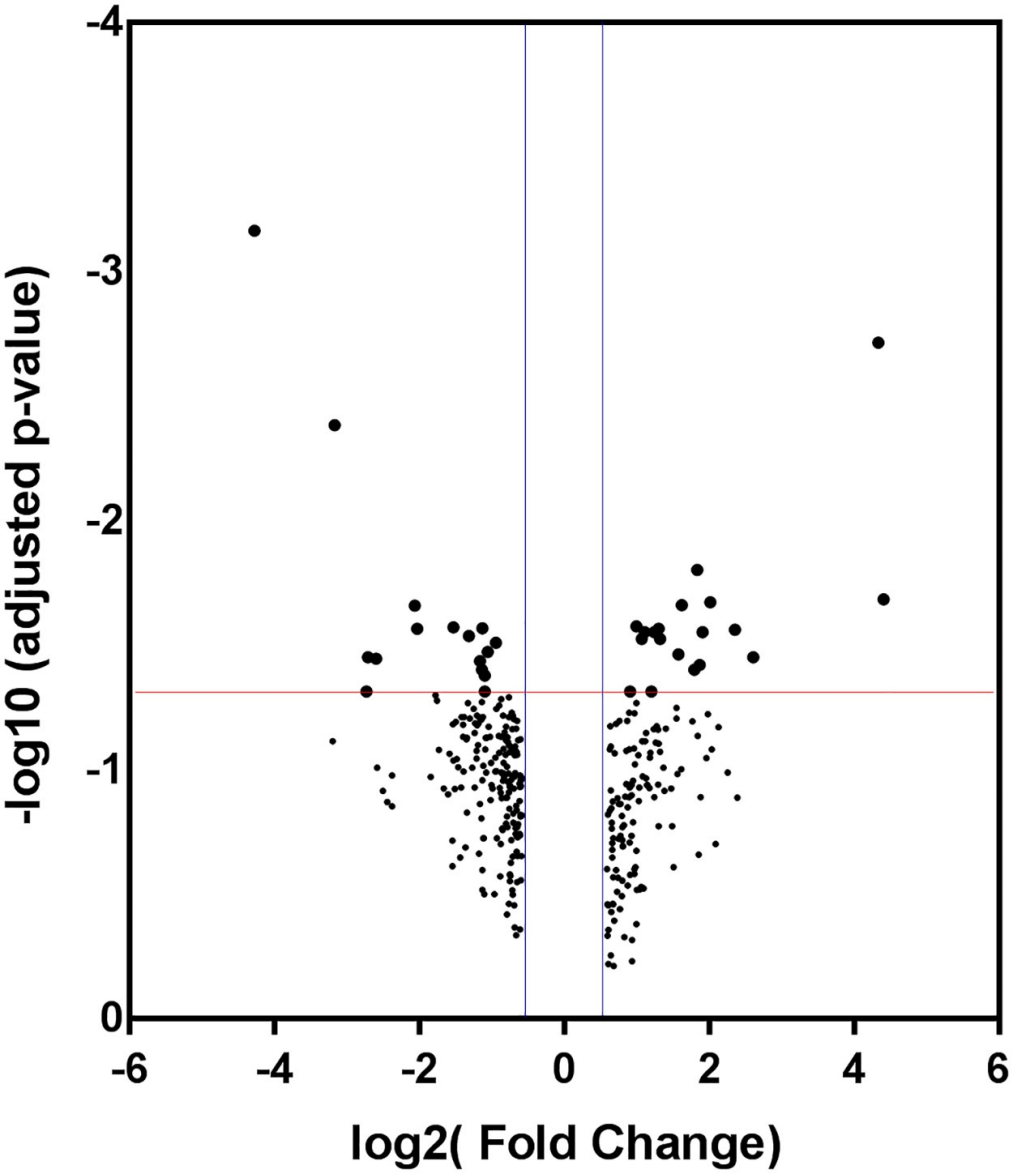
Volcano plot of the 342 lncRNAs identified with a fold-change > = 1.5. The red line represents the corrected p-value, which was set as cut-off (0.05). Positive values on the horizontal axis comprise up-regulated transcripts, and negative values on the same axis represent down-regulated lncRNAs, vertical lines at ~0.585 corresponding to 1.5 FC.

In the present study, we focused on the identification of long non-coding RNAs differentially expressed in TNBC. The analysis of the GSE98931 dataset revealed a total of 35 lncRNAs as differentially expressed in TNBC tumors (Fig 2, above the red line).

To access the potential prognostic value of the 35 lncRNAs identified as differentially expressed, we conducted survival analysis using the KM Plotter platform. Ten out of these lncRNAs (5 up and 5 down-regulated) were not found in the KM Plotter platform and could not be evaluated. For the other lncRNAs, we focused on those with significant associations with the survival rates of patients with breast tumors of the basal subtype, which best represents the triple-negative group of tumors. A total of 17 lncRNAs, 7 of which were up-regulated and 10 down-regulated, were associated with worse relapse-free survival in patients with the basal subtype (S1 Table).

Regarding overall survival, we identified 10 lncRNAs associated with worse prognosis in breast cancer patients with tumors of the basal subtype, of which 4 were up-regulated and 6 down-regulated in our cDNA microarray data (S2 Table). Up- and down-regulated lncRNAs identified as associated with poor prognosis in breast cancer patients with tumors of the basal subtype are summarized in Table 1. Table 1 also shows data reported in the literature classifying these lncRNAs as oncogene or tumor suppressor genes.

**Table 1:** Up and down-regulated lncRNAs associated with worse relapse-free survival (RFS) and overall survival (OS) in patients with basal breast cancer, according to the KM Plotter online platform and literature data.

LncRNA^OD^	Up-regulated		LncRNA^OD^	Down-regulated	
	Studies in breast cancer^RD^	Onco/TSG^RD^		Studies in breast cancer^RD^	Onco/TSG^RD^
***RFS—basal***					
LINC01018	No	TSG	LOC100130449	no	-
MIAT	Yes	Oncogene	MNX1-AS1	yes	Oncogene
AFAP1-AS1	Yes	Oncogene	LOC107984784	no	-
LINC00339	Yes	Oncogene	LOC285097	no	-
PAXIP1-AS1	No	-	LINC00548	no	-
LINC00869	No	-	CDKN2A-AS1	no	-
LOC729683	No	-	LINC00494	no	-
			LOC100130691	no	-
			WDFY3-AS2	no	TSG
			PRDM16-DT	no	TSG
***OS—basal***					
LINC00605	No	-	MNX1-AS1	yes	Oncogene
LINC02610	No	-	LINC00548	no	-
MIAT	Yes	Oncogene	CDKN2A-AS1	no	-
BDNF-AS	No	TSG	LOC100130691	no	-
			WDFY3-AS2	no	TSG
			PRDM16-DT	no	TSG

OD, our data; RD, review data; TSG, Tumor suppressor Gene.

We further investigated the expression pattern of the 35 lncRNAs in TANRIC and TCGA databases. We downloaded the spreadsheet with lncRNA expression information on breast tumors directly from the TANRIC database, which contains a total of 12,727 annotated long noncoding RNAs. Twelve out of these 35 lncRNAs were excluded because they were not included in the TANRIC expression dataset; however, 18 lncRNA transcripts were found differentially expressed when comparing normal tissue and tumor tissue (p <0.05; S3 Table).

The clinicopathological features of the patients were downloaded from the TCGA and UCSC platform. Data from both databases were cross-referenced to obtain clinical information pertinent for subsequent analyses. Including data of the different subtypes of breast cancer according to the PAM50 classification (Basal, Luminal A, Luminal B, HER2+ and Normal-like).

**Fig 3:**
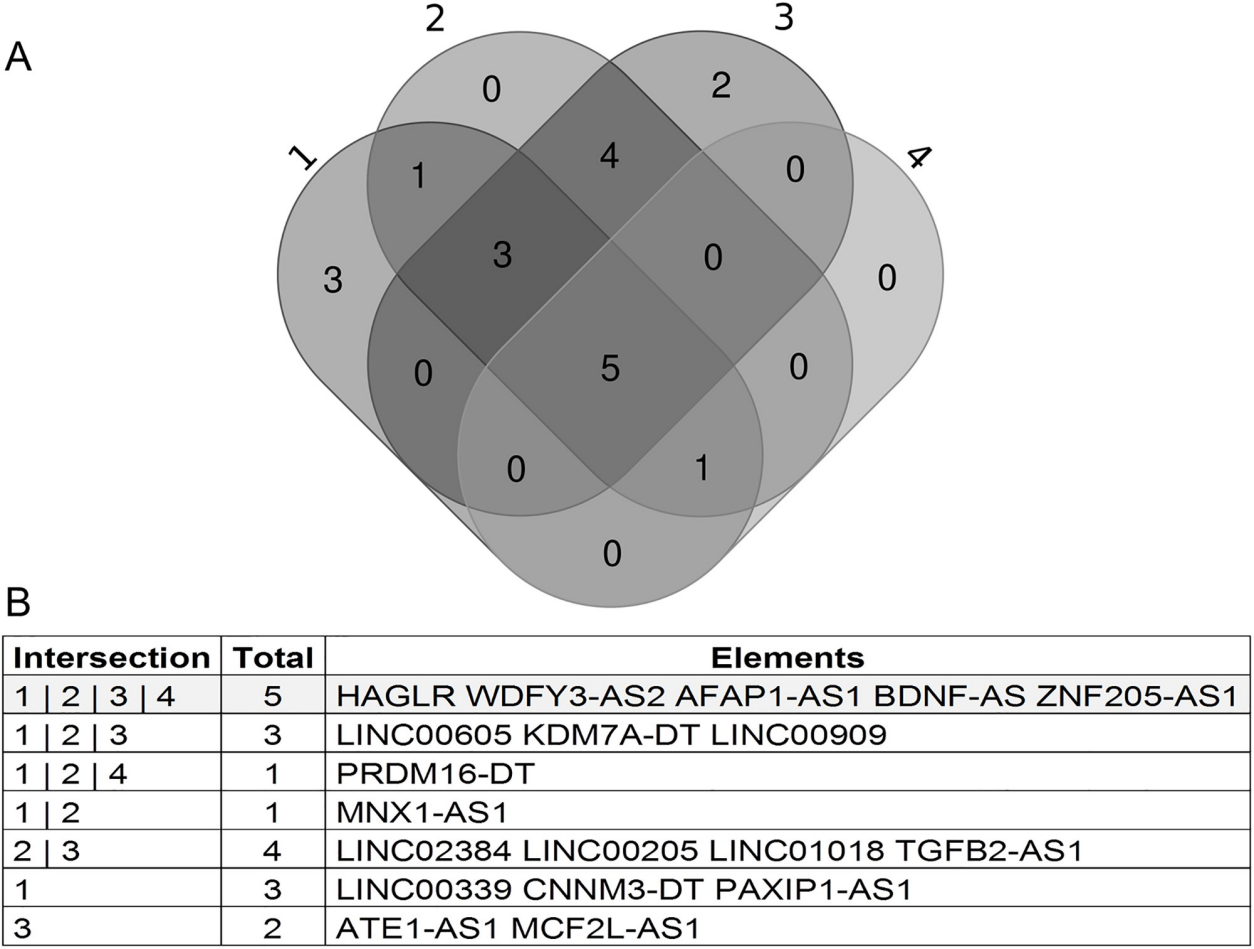
Venn diagram comparing basal subtype expression of lncRNAs relative to other breast cancer subtypes. A) Of the 35 differentially expressed lncRNAs identified in our microarray, 23 were found in the TANRIC database. These 23 transcripts were evaluated by the Mann-Whitney statistical test. The sets where the basal subtype is evidenced were used to construct the Venn diagram to identify transcripts differentially expressed primarily in basal tumors. A total of 5 transcripts, represented in the central intersection, were identified with a differential expression preferentially in basal tumors, among other subtypes. Set 1: Basal vs. HER2; set 2: Basal vs. Luminal A; set 3: Basal vs. Luminal B; set 4: Basal vs. Normal-like. B) Elements in the central intersection.

The Kruskal-Wallis test was used to identify differences in the expression pattern of 23 lncRNAs among the intrinsic tumor subtypes of the PAM50 classification. Statistically significant differences were found for a total of 21 lncRNAs (S4 Table). These 21 lncRNAs were further evaluated by the Mann-Whitney test and multiple comparisons in order to find transcripts with differential expression in the basal subtype compared to the other subtypes (S5 Table). Four sets of lncRNAs were established for the comparisons: set 1, concerns to transcripts with a p-value less than 0.005 in the Basal vs. HER2 + comparison; set 2, Basal vs. Luminal A; set 3, Basal vs. Luminal B; and set 4, Basal vs. Normal-like. The four sets of tlncRNAs transcripts were used to construct a Venn Diagram, where the central intersection represents the transcripts differentially expressed in the basal subtype compared to the other breast cancer subtypes (Fig 3).

The lncRNAs WDFY3-AS2, BDNF-AS, and AFAP1-AS1, identified as differentially expressed in basal tumors, were associated with poor survival and selected for subsequent analyses. WDFY3-AS2, BDNF-AS, and AFAP1-AS1 were classified into low or high expression according to the median expression value. These data were used to construct the contingency table according to the clinicopathological features of 828 patients coming from the TANRIC-TCGA platform. A descriptive analysis of the analyzable cases is presented in S6 and S7 Tables. In the analysis with all breast cancer subtypes, we observed significant differences in the status of hormone receptors (RP, p <0.0001; RE, p <0.0001), HER2 (p <0.0001), and PAM50 classification (p <0.0001) for those three lncRNAs. For the basal subtype tumors (139 cases), statistically significant differences were found regarding the patient’s age and BDNF-AS expression (p = 0.038) and AFAP1-AS1 (p = 0.011) (Table 2).

**Table 2:** Associations of WDFY-AS2, BDNF-AS, and AFAP1-AS1 expression and clinicopathological data of patients with basal subtype breast cancer from the TCGA-TANRIC bank.

Parameters	WDFY3-AS2			BDNF-AS			AFAP1-AS1		
	High	Low	*p-value*	High	Low	*p-value*	High	Low	*p-value*
**Age, n (%)**									
<50	9 (17,6)	42 (82,4)	0,525	20 (39,2)	31 (60,8)	0,038*	40 (93,0)	3 (7,0)	0,011*
>50	12 (13,6)	76 (86,4)		20 (22,7)	68 (77,3)		48 (71,6)	19 (28,4)	
**Race**									
Asian	0 (0.0)	7 (100,0)	0,443	3 (42,9)	4 (57,1)	0,400	5 (100,0)	0 (0,0)	0,651
Black or african american	3 (13,6)	19 (86,4)		4 (18,2)	18 (81,8)		16 (88,9)	2 (11,1)	
White	18 (17,6)	84 (82,4)		29 (28,4)	73 (71,6)		63 (78,8)	17 (21,3)	
**Primary diagnosis**									
Infiltrating duct carcinoma	18 (14,8)	104 (85,2)	0,612	36 (29,5)	86 (70,5)	0,806	77 (80,2)	19 (19,8)	0,148
Lobular carcinoma	0 (0.0)	3 (100,0)		1 (33,3)	2 (66,7)		1 (33,3)	2 (66,7)	
Other	3 (21,4)	11 (78,6)		3 (21,4)	11 (78,6)		10 (90,9)	1 (9,1)	
**TNM (T)**									
T1/T2	18 (14,9)	103 (85,1)	0,724	34 (28,1)	87 (71,9)	0,573	78 (80,4)	19 (19,6)	0,607
T3/T4	3 (17,6)	14 (82,4)		6 (35,3)	11 (64,7)		9 (75,0)	3 (25,0)	
**TNM (N)**									
N0	14 (16,1)	73 (83,9)	0,675	26 (29,9)	61 (70,1)	0,709	62 (84,9)	11 (15,1)	0,069
N1/N2/N3	7 (13,5)	45 (86,5)		14 (26,9)	38 (73,1)		26 (70,3)	11 (29,7)	
**TNM (M)**									
M0	21 (16,5)	106 (83,5)	1,000	34 (26,8)	93 (73,2)	0,185	80 (79,2)	21 (20,8)	0,692
M1	0 (0.0)	3 (100,0)		2 (66,7)	1 (33,3)		2 (100,0)	0 (0,0)	
**Stage**									
I/II	16 (14,2)	97 (85,8)	0,753	32 (28,3)	81 (71,7)	1,000	72 (80,0)	18 (20,0)	0,861
III/IV	4 (16,7)	20 (83,3)		7 (29,2)	17 (70,8)		14 (77,8)	4 (22,2)	
**Radiotherapy**									
No	9 (17,6)	42 (82,4)	0,701	17 (33,3)	34 (66,7)	0,291	33 (82,5)	7 (17,5)	0,889
Yes	11 (15,1)	62 (84,9)		18 (24,7)	55 (75,3)		48 (85,7)	8 (14,3)	
**Survival status**									
Alive	18 (14,9)	103 (85,1)	0,736	34 (28,1)	87 (71,9)	0,781	76 (80,0)	19 (20,0)	0,896
Dead	3 (16,7)	15 (83,3)		6 (33,3)	12 (66,7)		12 (80,0)	3 (20,0)	

*Significant values. Chi-square test or Fisher's exact test were applied as appropriate. For the analysis of the tumor stage we disregarded the group “Stage X” due to the small number of case.

**Fig 4:**
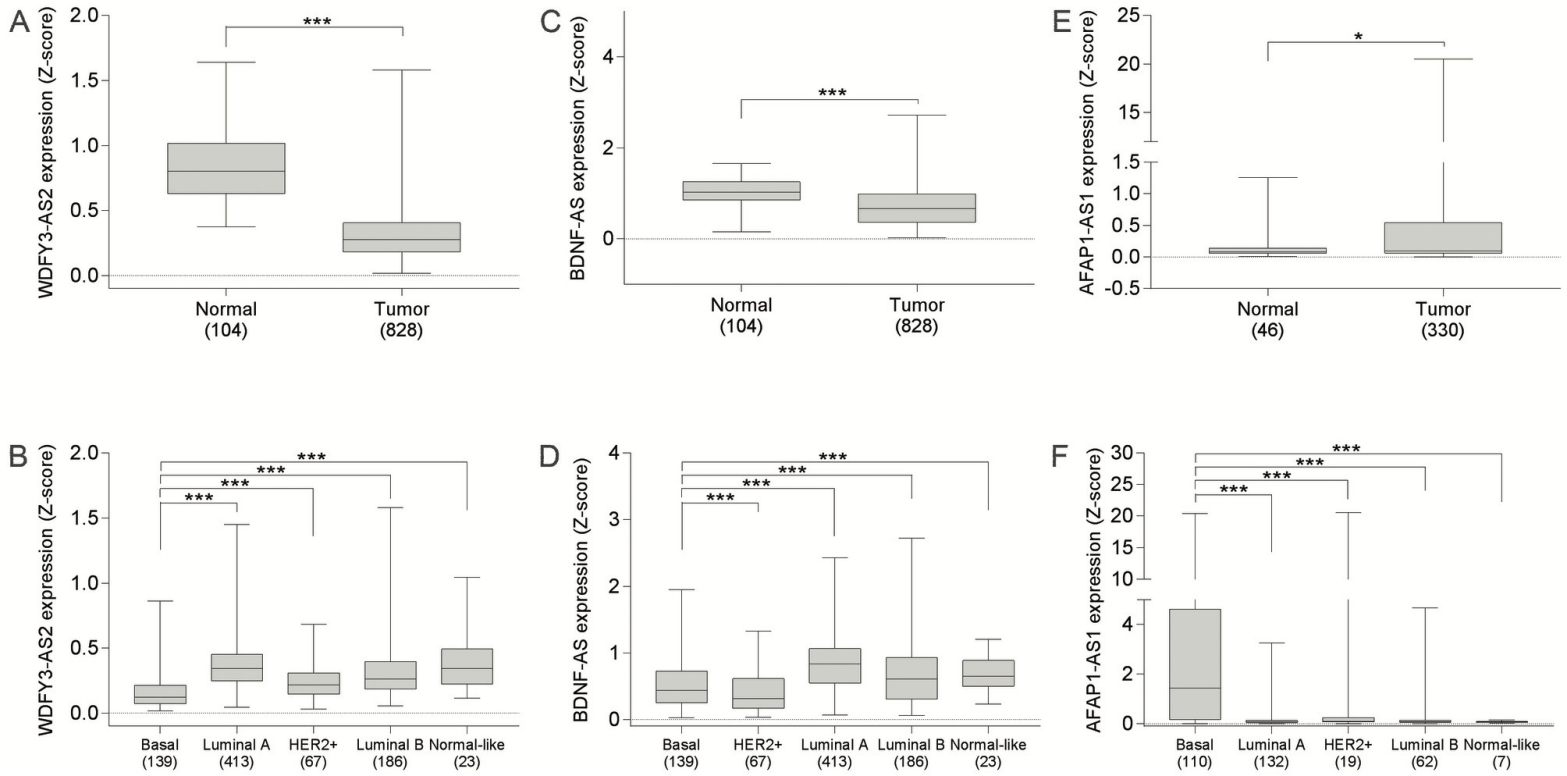
Expression pattern of WDFY3-AS2, BDNF-AS, and AFAP1-AS1 transcripts in breast cancer. Expression in normal versus tumor tissue (A, C, E); Expression pattern of WDFY3-AS2, BDNF-AS, and AFAP1-AS1 in different subtypes of breast cancer (B, D, F).

**Fig 5:**
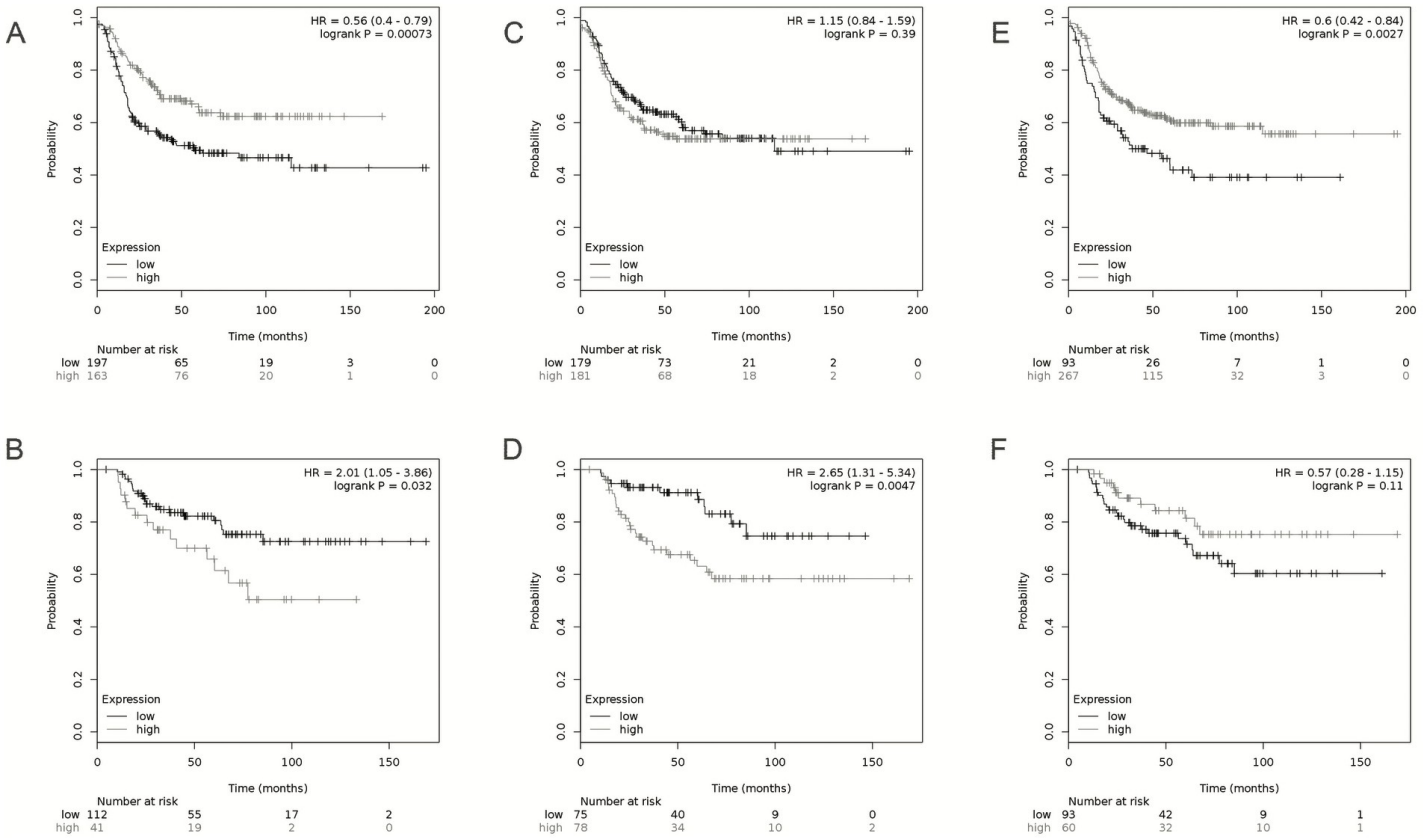
Kaplan-Meier of breast cancer patients stratified, according to WDFY3-AS2, BDNF-AS, and AFAP1-AS1 expression. Relapse-free survival for patients stratified by the expression of WDFY3-AS2 (A), BDNF-AS (C), and AFAP1-AS1 (E); Overall survival of patients stratified by the expression. WDFY3-AS2 (B), BDNF-AS (D), and AFAP1-AS1 (F). Survival analysis was conducted on the KM Plotter database (www.kmplot.com).

The long non-coding WDFY3-AS2 RNA was identified as downregulated in breast tumors relative to normal tissue (Fig 4A), and according to previous analyses in this study, low expression of this transcript was associated with worse prognosis in women with breast cancer (Fig 5A and 5B; Table 1; S1 and S2 Tables). Therefore, we conducted a multiple analysis (S5 Table) using the expression data from 828 patients and stratified the groups according to the PAM50 classification. The WDFY3-AS2 lncRNA was observed with the lowest expression in the basal subtype (Fig 4B).

Our analysis using cBioPortal databases showed that the WDFY3-AS2 lncRNA is positively correlated with the expression of WDFY3 (R = 0.7; S1A Fig). We further investigated WDFY3 expression in breast tumors from the UALCAN platform, and similar to WDFY3-AS2, low expression of WDFY3 was observed in these tumors (p = 1.7E-12; S2A Fig), being significantly low expressed in the triple-negative subtypes (p = 1,6E-12; S2B Fig). WDFY3 low expression was associated with poor overall and relapse-free survival in breast cancer patients with tumors of the Luminal subtype. However, the survival probabilities between patients with tumors of the basal subtype with low and high WDFY3 expression were not statistically significant (S3 Fig).

We identified BDNF-AS transcripts as down-regulated in breast tumors (Fig 4C). Regarding the PAM50 classification, low BDNF-AS expression was observed in the basal subtype (Fig 4D). The heat map of 104 cases of matched normal and tumor tissue visually shows us a reduced expression of BDNF-AS in normal tissue relative to tumor tissue (S4A Fig). Survival analysis showed that low BNDF-AS expression is associated with worse prognosis in patients with basal breast cancer (Fig 5C and 5D). A correlation analysis was conducted, and a significant result was found between the expression of BDNF-AS and BDNF (S4B Fig; R2 = 0.3; p <0.0001). Reduced BDNF expression was associated with worse disease-free survival and overall survival in breast cancer patients (TNBC, RFS, log-rank p = 0.0094; S5 Fig).

AFAP1-AS1 was observed with high expression in breast tumors compared to the normal tissue (Fig 4E). Regarding the PAM50 classification, AFAP1-AS1 overexpression was observed in the basal subtype compared to the other subtypes (Fig 4F). Survival analysis showed that low AFAP1-AS1 expression is associated with a worse prognosis in patients with basal breast cancer (Fig 5E and 5F). We conducted a correlation analysis with paired data from TANRIC-cBioPortal databases, and statistically significant associations were found (S6 Fig; R2 = 0.33; p <0.001). Survival curves obtained by the KM Plotter platform show that increased expression of AFAP1 transcripts is associated with short disease-free interval in patients with Luminal B (p = 0.0057), HER2 + (p = 0.017), and basal (p = 0.0052) tumors and worse overall survival Luminal A (0.02) and HER2 + (p <0.0001) tumor subtypes.

## Discussion

In the present study, we sought to identify differentially expressed lncRNAs from a microarray data set available in the GEO database, generated in a previous study from our group [42]. The differentially expressed lncRNAs were further evaluated using different databases to assess their potential prognostic value for breast cancer patients. The transcripts of three lncRNAs stood out in our analysis, namely WDFY3-AS2, AFAP1-AS1, and BDNF-AS, for showing differential expression in the basal subtype compared to the other subtypes of the PAM50 classification.

Downregulation of WDFY3-AS2 expression has been reported in different types of tumors, including breast cancer [50–52]. WDFY3-AS2 was found down-regulated in glioma and was associated with poor patient survival [51]. An *in silico* study identified WDFY3-AS2 as down-regulated and associated with a worse prognosis in esophageal cancer [50]. WDFY3-AS2 knockdown resulted in decreased expression of N-cadherin in liver carcinoma cell lines, suggesting that WDFY3-AS2 is associated with the process of epithelial-mesenchymal transition (EMT) in hepatocellular carcinoma (HCC) cells [53]. These authors also demonstrated a significant decrease in the invasive and migratory capability of HCC cells after WDFY3-AS2 knockdown [53]. In ovarian carcinoma, Li et al. [54] identified reduced WDFY3-AS2 expression in tumor tissue compared to adjacent normal tissue [54]. They also demonstrated that WDFY3-AS2 overexpression led to inhibition of cell proliferation, migration, and invasion and decreased protein levels of N-cadherin and increased E-cadherin expression [54]. Recently, Deva Magendhra Rao et al. [52], using RNA sequencing to determine the expression profile of lncRNA in breast cancer, identified WDFY3-AS2 as down-regulated in early-stage breast tumors, which was validated in 52 tumor samples. Performing data mining on the TCGA dataset, these authors also found that altered expression of WDFY3-AS2 transcripts is associated with a worse prognosis for breast cancer patients [52]. Here, we found that low WDFY3-AS2 expression is associated with worse prognosis in breast cancer patients, including those classified as a basal or triple-negative subtype, suggesting that WDFY3-AS2 may act as a tumor suppressor gene for breast cancer. Those data provide new insights on the role played by WDFY3-AS2 in the tumorigenic process, including TNBC breast tumors, which warranted furthers experimental and clinical studies to better understand the potential role of WDFY3-AS2 as a breast cancer biomarker.

BDNF-AS is a tumor suppressor originally described as antisense to the brain-derived neurotrophic factor (BDNF) gene [55–57]. Zhi and Lian [57] identified reduced expression of BDNF-AS in colorectal tumors, and they demonstrated that BDNF-AS overexpression inhibited proliferation and decrease the migratory and invasive capability of LoVo colon cancer cell [57]. On the other hand, BDNF-AS silencing in HCT116 cells resulted in increased proliferation, migration, and invasion, suggesting that BDNF-AS acts as a tumor suppressor in colorectal cancer. The authors also found that BDNF-AS works by regulating the expression of GSK-3β –a known oncogene [57]. BDNF-AS reduced expression was found in osteosarcoma and was associated with worse prognosis in a cohort of 114 patients [55]. HUANG et al. [55] also demonstrated that BDNF-AS overexpression results in the inhibition of proliferation in osteosarcoma cells. A study published by Li et al. [58] identified the down-regulation of BDNF-AS in a cohort of 141 prostate cancer patients [58]. Overexpression of BDNF-AS transcripts in PSA negative and PSA positive prostate cancer cell lines (PC-3 and LNCaP, respectively) resulted in a lower proliferative rate and significantly decreased the invasive capability of prostate cancer cells [58]. In the present study, we found that BDNF-AS transcripts were significantly down-regulated in breast tumors compared with normal tissue, and we further revealed that low BNDF-AS expression is associated with worse prognosis in patients with basal breast cancer subtype. However, further clinical and experimental studies are needed to elucidate the biologic and clinical significance of BDNF-AS expression in breast cancer before to define the clinical utility of this potential biomarker.

The lncRNA AFAP1-AS1 (actin filament associated protein 1 antisense RNA 1) overlaps the exons 2, 14, 15, and 16 of the AFAP1 gene and is the most investigated among lncRNAs found in our study. AFAP1-AS1 is reported in the literature as an oncogene, including in triple-negative breast cancer [59, 60]. In the study by Zhang et al. [53], AFAP1-AS1 up-regulation was associated with TNBC. Analysis of overall survival and disease-free survival in a cohort of 238 patients undergoing mastectomy and chemotherapy revealed that AFAP1-AS1 overexpression was associated with poor prognosis in TNBC patients. In vitro assays, demonstrated that AFAP1-AS1 knockdown resulted in reduced proliferation and invasion and increased apoptosis rates in MDA-MB-231 and BT-549 cell lines [60]. In vivo, overexpression of AFAP1-AS1 promoted tumor growth and activated Wnt/β-catenin pathway to promote tumorigenesis and cell invasion by increasing the expression of C-myc and EMT related genes [60]. Here, we also found overexpression of AFAP1-AS1 in the basal subtype compared to the other breast cancer subtypes; however, our in silico survival analysis revealed that low AFAP1-AS1 expression is associated with a worse prognosis in patients with breast cancer of the basal subtype. These findings imply that altered expression of AFAP1-AS1 transcripts is associated with tumorigenesis in TNBC cancer; however, the role played by AFAP1-AS1 in the tumorigenic process of TNBC remains to be further verified by the larger sample scale.

Our study was able to identify three lncRNAs as potential biomarkers in breast cancer, and which functions have not been already fully understood or elucidated in the context of breast cancer. Although preliminary, our *in silico* results are promising, and further clinical and functional assays are necessary to understand the particularities of the cellular effect achieved at the expense of modulating the expression of these transcripts, as well as their interaction with other biomolecules, such as microRNAs and transcription factors.

## Supporting information

S1 Fig WDFY3-AS2 expression in breast cancer. A) correlation analysis between WDFY3 and WDFY3-AS2 expression z-score. Analyses were performed using 1108 cases of breast tumors from cBioPortal. B) Heat Map with paired cases of 104 samples from the TANRIC database.
(JPG)

S2 Fig WDFY3 expression in breast cancer. A) Box plot showing the WDFY3 expression between normal and tumor tissue. B) WDFY3 expression level in breast cancer subtypes. Charts from the UALCAN online platform.
(TIFF)

S3 Fig Relapse-free survival and overall survival for patients stratified by the expression of WDFY3. A, RFS for all breast cancer patients; B, RFS for TNBC patients; C, OS for all breast cancer patients; D, OS for TNBC patients.
(JPG)

S4 Fig BDNF-AS expression in breast cancer. A) correlation analysis between BDNF and BDNF-AS expression z-score. Analyses were performed using 1108 cases of breast tumors from cBioPortal. B) Heat Map with paired cases of 104 samples from the TANRIC database.
(JPG)

S5 Fig Relapse-free survival and overall survival for patients stratified by the expression of BNDF. A, RFS for all breast cancer patients; B, RFS for TNBC patients; C, OS for all breast cancer patients; D, OS for TNBC patients.
(JPG)

S6 Fig Correlation analysis between AFAP1 and AFAP1-AS1 expression z-score. Analyses were performed using 1108 cases of breast tumors from cBioPortal.
(PNG)

S1 Table Association of lncRNAs expression with relapse-free survival in breast cancer patients. (DOCX)

S2 Table Association of lncRNAs expression with overall survival in breast cancer patients. (DOCX)

S3 Table Statistical analysis comparing lncRNA expression differences between normal tissue (104 cases) and tumor tissue (828 cases). (DOCX)

S4 Table Statistical analysis comparing differences in lncRNA expression between intrinsic subtypes of the PAM50 classification. (DOCX)

S5 Table Multiple analysis comparing lncRNAs expression in different breast cancer subtypes. (DOCX)

S6 Table Frequency of cases by lncRNA according to expression data available from TANRIC database. (DOCX)

S7 Table Clinicopathological data of breast cancer patients from TANRIC-TCGA bank. (DOCX)
