# Supplementary figures and images for: In silico analyses identify lncRNAs: WDFY3-AS2, BDNF-AS and AFAP1-AS1 as potential prognostic factors for patients with triple-negative breast tumors
Source: PLoS One. 2020 May 13;15(5):e0232284. doi: 10.1371/journal.pone.0232284 (PMC7219740; doi:10.1371/journal.pone.0232284)

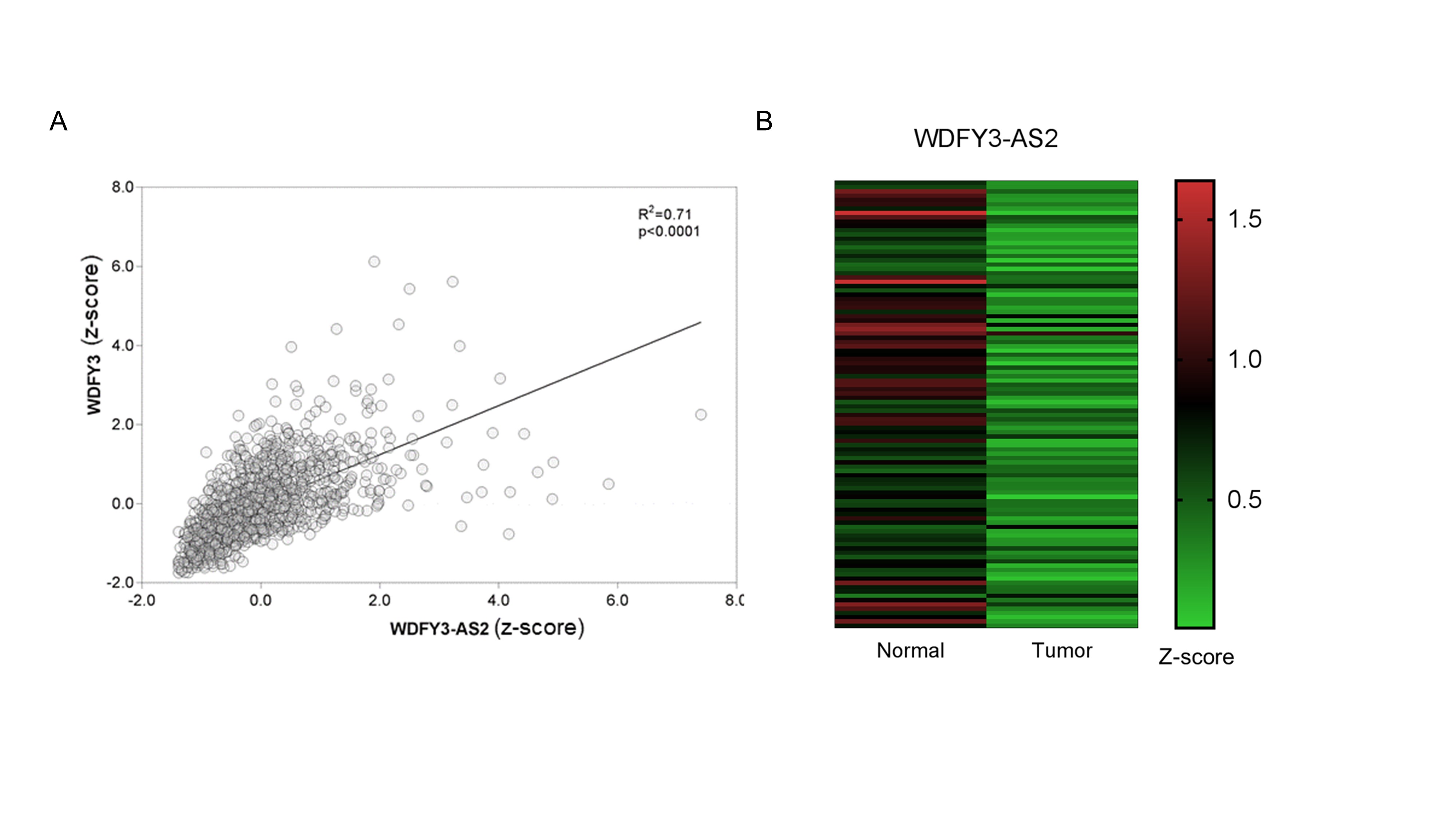

Supplement: S1 Fig — A) correlation analysis between WDFY3 and WDFY3-AS2 expression z-score. Analyses were performed using 1108 cases of breast tumors from cBioPortal. B) Heat Map with paired cases of 104 samples from the TANRIC database. (JPG) [file pone.0232284.s001.jpg]

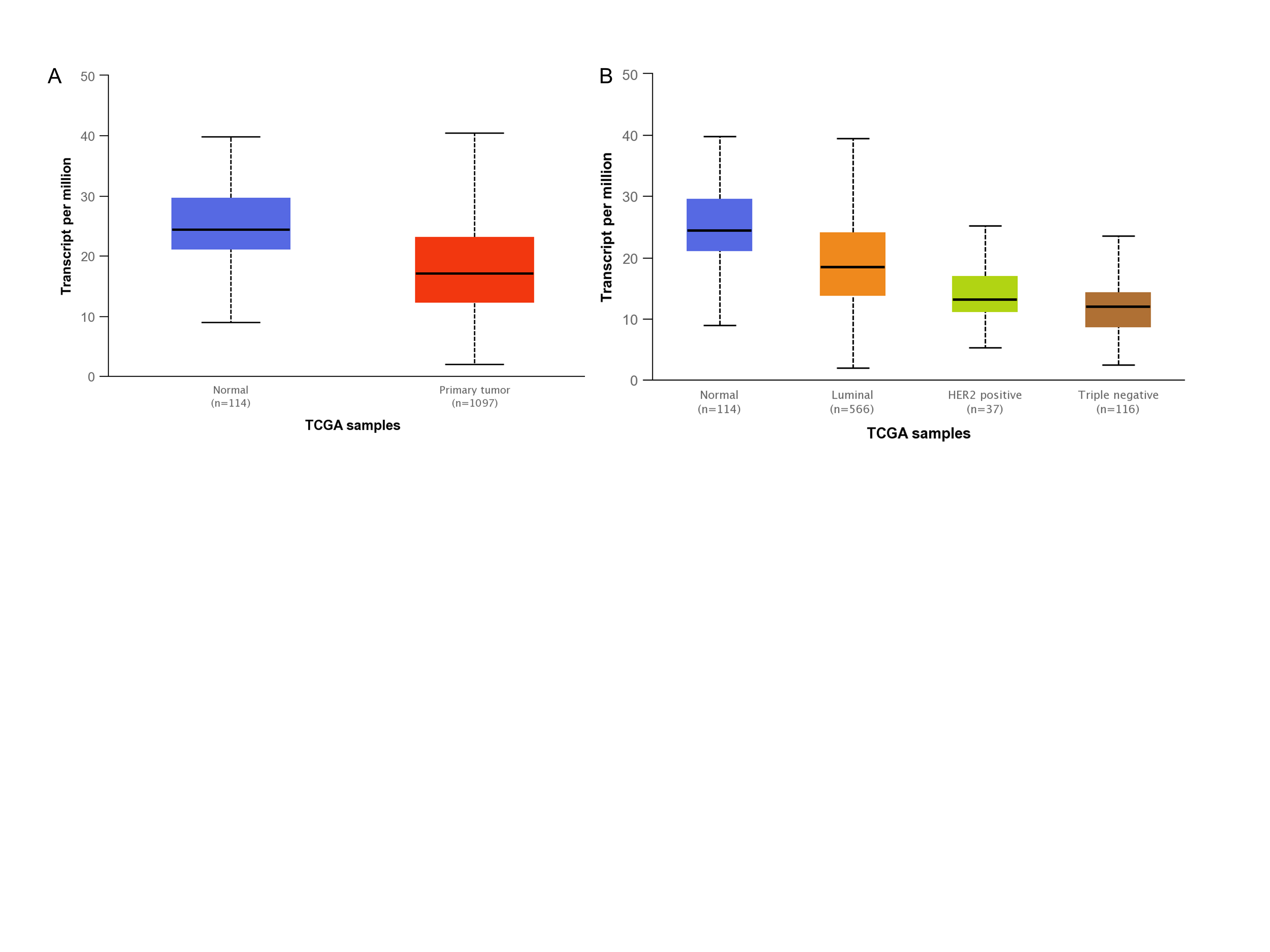

Supplement: S2 Fig — A) Box plot showing the WDFY3 expression between normal and tumor tissue. B) WDFY3 expression level in breast cancer subtypes. Charts from the UALCAN online platform. (TIFF) [file pone.0232284.s002.tiff]

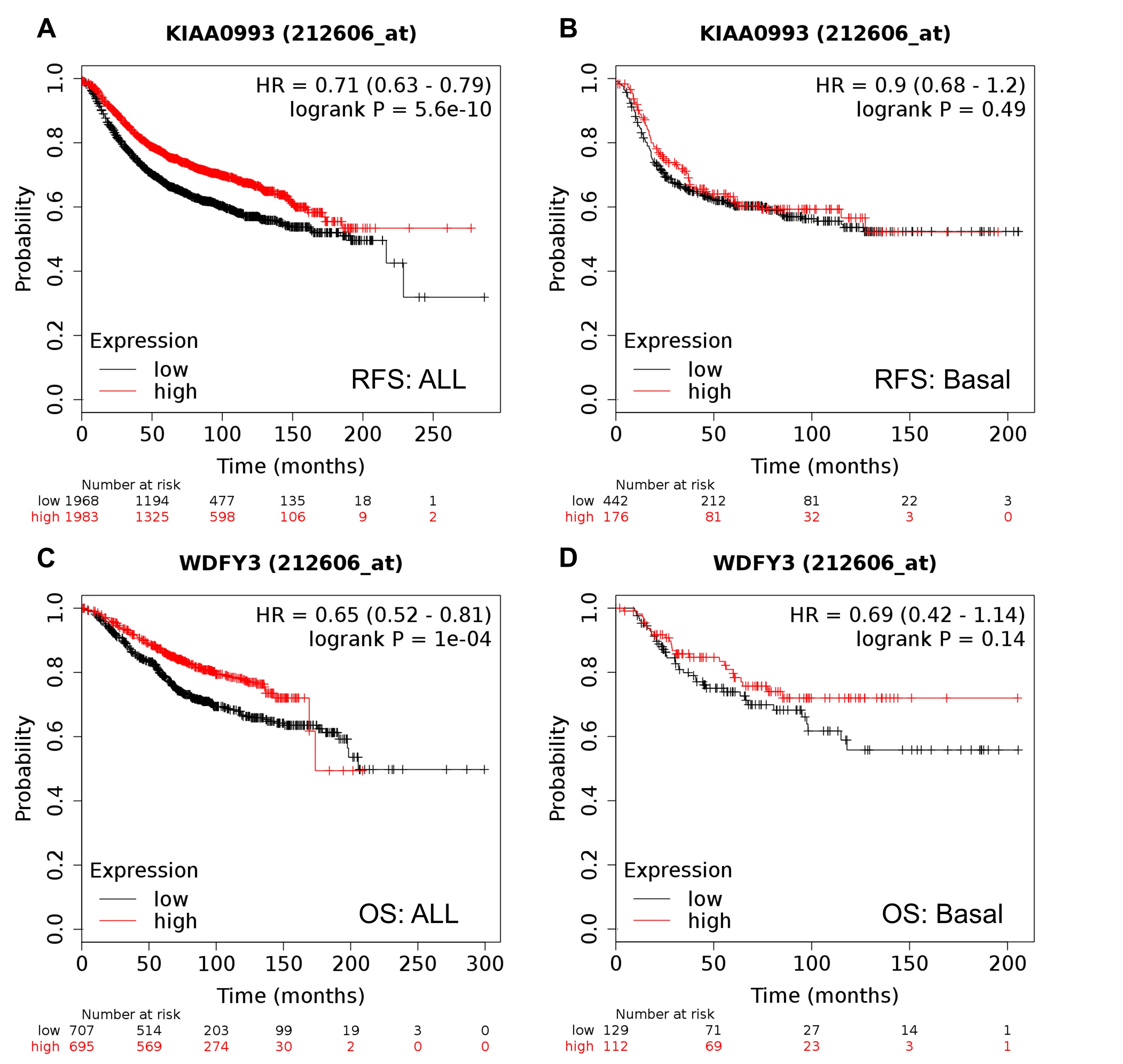

Supplement: S3 Fig — A, RFS for all breast cancer patients; B, RFS for TNBC patients; C, OS for all breast cancer patients; D, OS for TNBC patients. (JPG) [file pone.0232284.s003.jpg]

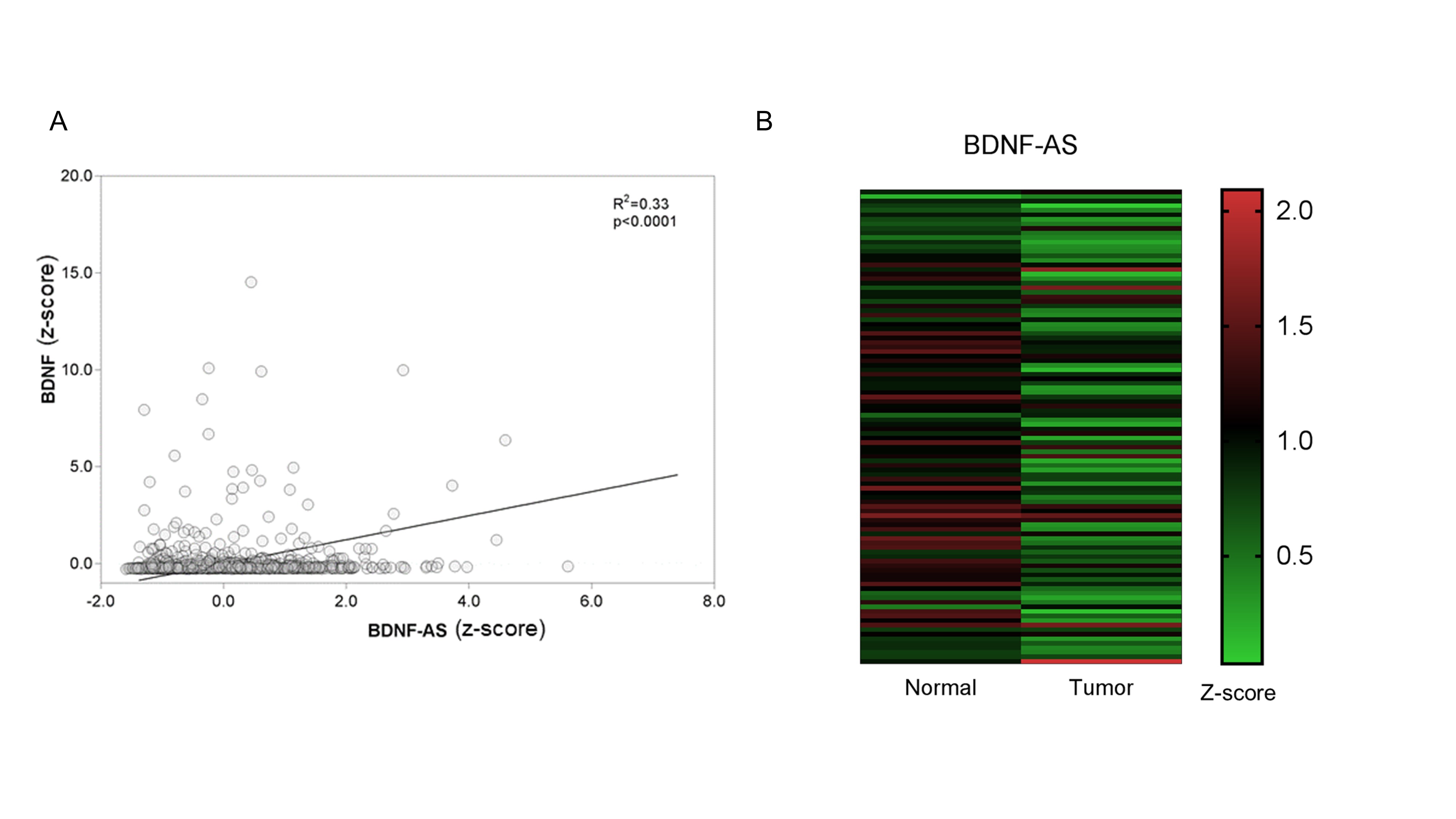

Supplement: S4 Fig — A) correlation analysis between BDNF and BDNF-AS expression z-score. Analyses were performed using 1108 cases of breast tumors from cBioPortal. B) Heat Map with paired cases of 104 samples from the TANRIC database. (JPG) [file pone.0232284.s004.jpg]

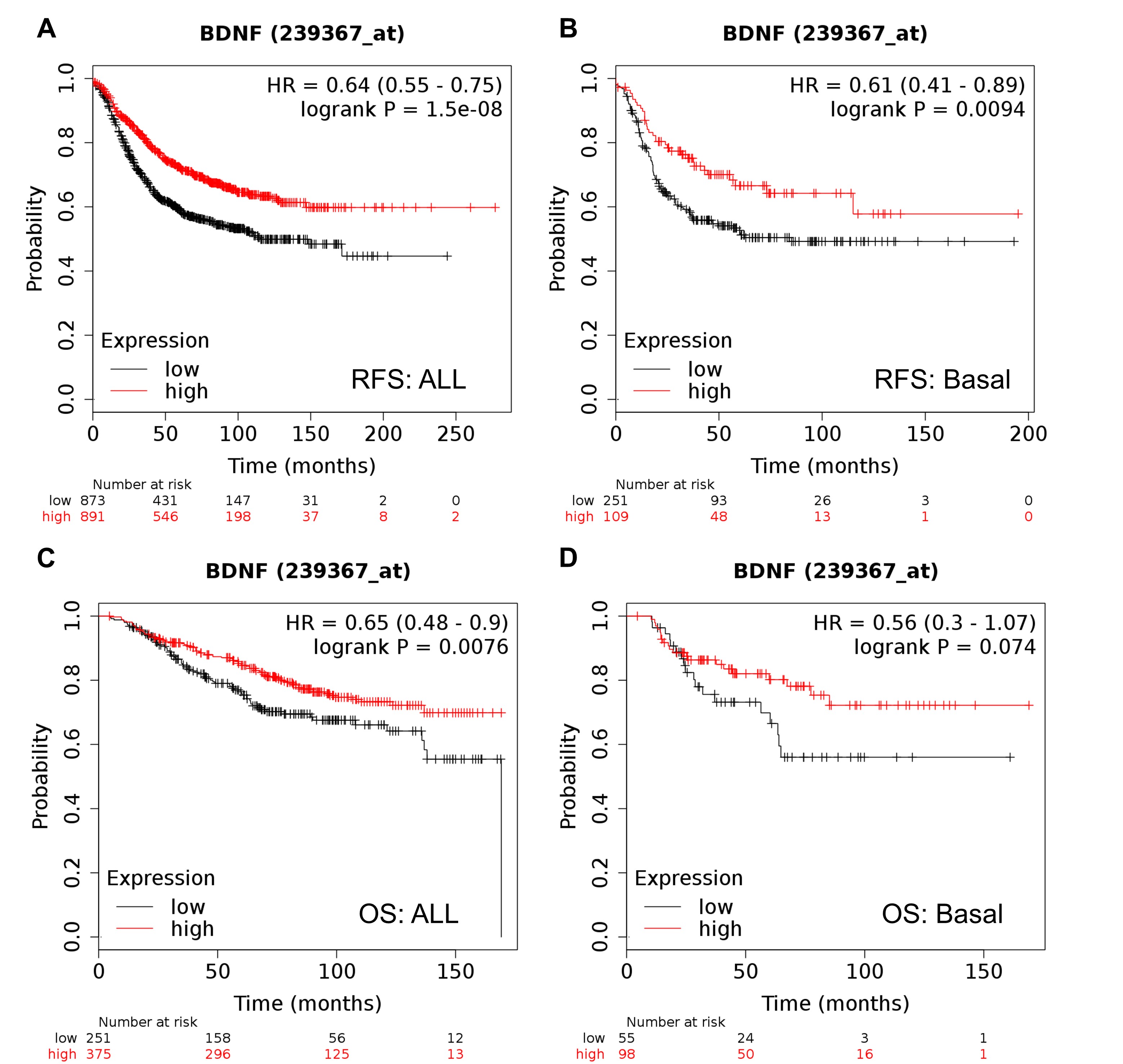

Supplement: S5 Fig — A, RFS for all breast cancer patients; B, RFS for TNBC patients; C, OS for all breast cancer patients; D, OS for TNBC patients. (JPG) [file pone.0232284.s005.jpg]

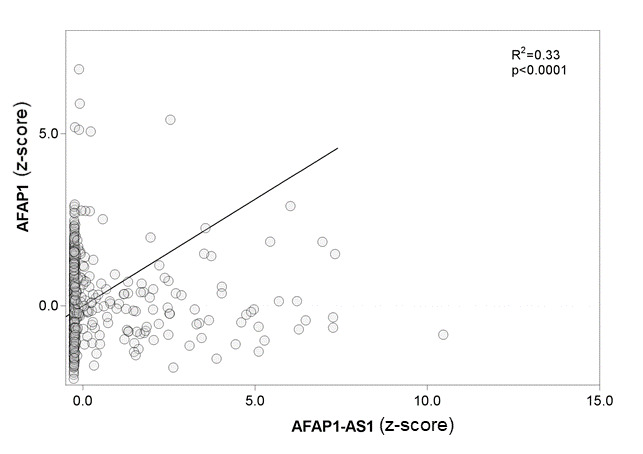

Supplement: S6 Fig — Analyses were performed using 1108 cases of breast tumors from cBioPortal. (PNG) [file pone.0232284.s006.png]
