# Supplementary material for: In silico analyses identify lncRNAs: WDFY3-AS2, BDNF-AS and AFAP1-AS1 as potential prognostic factors for patients with triple-negative breast tumors
Source: PLoS One. 2020 May 13;15(5):e0232284. doi: 10.1371/journal.pone.0232284 (PMC7219740; doi:10.1371/journal.pone.0232284)
Supplement: S1 Table — (DOCX) [file pone.0232284.s007.docx]

**Suppl. Table 1 -** Association of lncRNAs expression with relapse-free survival in breast cancer patients.

| **lncRNA** | **All subtypes** | **Expression*** | **Basal** | **Expression*** | **Luminal A** | **Expression*** | **Luminal B** | **Expression*** | **HER2+** | **Expression*** |
| --- | --- | --- | --- | --- | --- | --- | --- | --- | --- | --- |
| **UP** | | | | | | | | | | |
| LINC01018 | 3,20E-09 | Low | 4,80E-07 | Low | 0,00014 | Low | 0,0013 | Low | 0,09 | No difference |
| TGFB2-AS1 | - | - | - | - | - | - | - | - | - | - |
| LINC00605 | 0,13 | No difference | 0,23 | No difference | 0,5 | No difference | 0,22 | No difference | 0,0083 | High |
| ATE1-AS1 | - | - | - | - | - | - | - | - | - | - |
| MCF2L-AS1 | - | - | - | - | - | - | - | - | - | - |
| LOC101928424 | 1,20E-07 | Low | 0,1586 | No difference | 9,30E-08 | Low | 0,0031 | Low | 0,1602 | No difference |
| LINC02384 | - | - | - | - | - | - | - | - | - | - |
| LINC02610 | 0.0002 | Low | 0.0738 | No difference | 0.0024 | Low | 0.0017 | Low | 0.1647 | No difference |
| MIAT | 2.2e-16 | Low | 4.2e-8 | Low | 6.2e-8 | Low | 7.8e-7 | Low | 0.0005 | Low |
| LINC00205 | 1.6e-14 | Low | 0.0637 | No difference | 5.7e-10 | Low | 2.6e-5 | Low | 0.001 | Low |
| AFAP1-AS1 | 2.5e-7 | Low | 0.0027 | Low | 1.9e-6 | Low | 0.0028 | Low | 0.0013 | Low |
| LINC00909 | 0.0002 | Low | 0.1026 | No difference | 0.0269 | Low | 0.0264 | Low | 0.0085 | Low |
| KDM7A-DT | 0.4523 | No difference | 0.1158 | No difference | 0.1715 | No difference | 0.0004 | Low | 0.2881 | No difference |
| LINC00339 | 0.0071 | Low | 0.0116 | High | 0.1351 | No difference | 0.1997 | No difference | 0.011 | Low |
| PAXIP1-AS1 | <1e-16 | Low | 0.0044 | Low | 4.1e-11 | Low | 0.0026 | Low | 0.2595 | No difference |
| LINC00869 | 3.2e-10 | Low | 6.1e-7 | Low | 3.1e-5 | Low | 0.004 | Low | 0.0242 | Low |
| BDNF-AS | 0.0007 | Low | 0.3861 | No difference | 0.0016 | Low | 0.1497 | No difference | 0.0731 | No difference |
| LOC729683 | 4.8e-6 | Low | 4.5e-5 | Low | 0.0063 | Low | 0.0181 | Low | 0.0956 | No difference |
| CNNM3-DT | - | - | - | - | - | - | - | - | - | - |
| **DOWN** | | | | | | | | | | |
| LOC101929056 | - | - | - | - | - | - | - | - | - | - |
| LOC100130449 | 7.9e-5 | Low | 0.0013 | Low | 0.0059 | High | 0.0908 | No difference | 0.3326 | No difference |
| MNX1-AS1 | 3.0e-10 | High | 0,0106 | High | 3.9e-5 | High | 0.008 | High | 0.0496 | High |
| LOC107984784 | 6.2e-5 | Low | 0.0065 | Low | 0.0012 | Low | 0.0152 | Low | 0.1118 | No difference |
| LOC285097 | 6.8e-11 | Low | 6.6e-6 | Low | 1.5e-8 | Low | 0.1135 | No difference | 0.0381 | High |
| LOC100128340 | - | - | - | - | - | - | - | - | - | - |
| LINC00548 | 1.4e-12 | Low | 5.6e-6 | Low | 1.2e-7 | Low | 0.0023 | Low | 0.1393 | No difference |
| CDKN2A-AS1 | 0.0665 | No difference | 0.0106 | High | 0.0878 | No difference | 0.0404 | Low | 0.012 | High |
| HAGLR | 0.0216 | High | 0.1037 | No difference | 0.2714 | No difference | 0.021 | High | 0.0451 | High |
| LINC00494 | 7.8e-6 | Low | 0.0048 | Low | 0.0202 | Low | 5,00E-04 | Low | 0.1088 | No difference |
| ZNF205-AS1 | - | - | - | - | - | - | - | - | - | - |
| LOC100130691 | <1e-16 | Low | 6.7e-7 | Low | 3.5e-8 | Low | 0.0005 | Low | 0.1197 | No difference |
| LINC01711 | - | - | - | - | - | - | - | - | - | - |
| LINC00618 | - | - | - | - | - | - | - | - | - | - |
| WDFY3-AS2 | 2.4e-15 | Low | 0.0007 | Low | 9.2e-8 | Low | 0.0046 | Low | 0.1211 | No difference |
| PRDM16-DT | 0.0263 | High | 0.0236 | High | 0.0262 | Low | 0.0091 | High | 0.032 | Low |

*Low or High or No difference lncRNA expression associated with worse survival.
