# Supplementary material for: In silico analyses identify lncRNAs: WDFY3-AS2, BDNF-AS and AFAP1-AS1 as potential prognostic factors for patients with triple-negative breast tumors
Source: PLoS One. 2020 May 13;15(5):e0232284. doi: 10.1371/journal.pone.0232284 (PMC7219740; doi:10.1371/journal.pone.0232284)
Supplement: S2 Table — (DOCX) [file pone.0232284.s008.docx]

**Suppl. Table 2** - Association of lncRNAs expression with overall survival in breast cancer patients.

| **lncRNA** | **All subtypes** | **Expression*** | **Basal** | **Expression*** | **Luminal A** | **Expression*** | **Luminal B** | **Expression*** | **HER2+** | **Expression*** |
| --- | --- | --- | --- | --- | --- | --- | --- | --- | --- | --- |
| **UP** | | | | | | | | | | |
| LINC01018 | 0,32 | No difference | 0,35 | No difference | 0,34 | No difference | 0,17 | No difference | 0,29 | No difference |
| TGFB2-AS1 | - | - | - | - | - | - | - | - | - | - |
| LINC00605 | 3,00E-06 | Low | 0,0029 | Low | 0,00069 | Low | 8,50E-06 | Low | 0,16 | No difference |
| ATE1-AS1 | - | - | - | - | - | - | - | - | - | - |
| MCF2L-AS1 | - | - | - | - | - | - | - | - | - | - |
| LOC101928424 | 0,0962 | No difference | 0,115 | No difference | 0,0425 | Low | 0,134 | No difference | 3,70E-05 | High |
| LINC02384 | - | - | - | - | - | - | - | - | - | - |
| LINC02610 | 0.0604 | No difference | 0.0177 | High | 0.3552 | No difference | 0.2016 | No difference | 0.285 | No difference |
| MIAT | 2.5e-5 | Low | 0.0239 | Low | 0.0002 | Low | 0.0279 | Low | 0.0732 | No difference |
| LINC00205 | 0.0429 | High | 0.099 | No difference | 0.022 | High | 0.4064 | Low | 0.0166 | High |
| AFAP1-AS1 | 0.2159 | No difference | 0.1134 | No difference | 0.0154 | Low | 0.2247 | No difference | 0.1592 | No difference |
| LINC00909 | 0.0049 | Low | 0.4534 | No difference | 0.2098 | No difference | 0.2673 | No difference | 0.001 | Low |
| KDM7A-DT | 0.0347 | High | 0.213 | No difference | 0.09 | No difference | 0.0322 | Low | 0.2113 | No difference |
| LINC00339 | 0.1734 | No difference | 0.1455 | No difference | 0.2837 | No difference | 0.2338 | No difference | 0.1462 | No difference |
| PAXIP1-AS1 | 0.0002 | Low | 0.0745 | No difference | 0.0042 | Low | 0.0228 | Low | 0.1363 | No difference |
| LINC00869 | 0.358 | No difference | 0.1232 | No difference | 0.2245 | No difference | 0.0013 | High | 0.0266 | High |
| BDNF-AS | 0.0649 | No difference | 0.0047 | High | 0.0017 | Low | 0.21 | No difference | 0.0015 | High |
| LOC729683 | 0.0237 | High | 0.0811 | No difference | 0.0193 | High | 0.0243 | High | 0.1077 | No difference |
| CNNM3-DT | - | - | - | - | - | - | - | - | - | - |
| **DOWN** | | | | | | | | | | |
| LOC101929056 | - | - | - | - | - | - | - | - | - | - |
| LOC100130449 | 0.0246 | High | 0.0676 | No difference | 0.0705 | No difference | 0.0395 | High | 0.2515 | No difference |
| MNX1-AS1 | 7.4e-6 | High | 0.0065 | High | 0.0369 | High | 0.0064 | High | 0.1269 | No difference |
| LOC107984784 | 0.0618 | No difference | 0.0651 | No difference | 0.0532 | No difference | 0.0992 | No difference | 0.0292 | High |
| LOC285097 | 0.0005 | Low | 0.1121 | No difference | 0.0004 | Low | 0.0228 | High | 0.0331 | High |
| LOC100128340 | - | - | - | - | - | - | - | - | - | - |
| LINC00548 | 0.0229 | Low | 0.0066 | Low | 0.0692 | No difference | 0.2241 | No difference | 0.015 | High |
| CDKN2A-AS1 | 0.0975 | No difference | 0.0003 | High | 0.0067 | Low | 0.0204 | Low | 0.0197 | High |
| HAGLR | 0.0862 | No difference | 0.096 | No difference | 0.3222 | No difference | 0.0054 | High | 0.354 | No difference |
| LINC00494 | 0.0207 | Low | 0.0562 | No difference | 0.3546 | No difference | 0.0056 | Low | 0.2393 | No difference |
| ZNF205-AS1 | - | - | - | - | - | - | - | - | - | - |
| LOC100130691 | 0.0112 | Low | 0.0065 | Low | 0.0357 | Low | 0.0602 | High | 0.0061 | High |
| LINC01711 | - | - | - | - | - | - | - | - | - | - |
| LINC00618 | - | - | - | - | - | - | - | - | - | - |
| WDFY3-AS2 | 0.1825 | No difference | 0.0316 | High | 0.1753 | No difference | 0.1224 | No difference | 0.009 | High |
| PRDM16-DT | 0.0116 | High | 0.0273 | High | 0.009 | High | 0.2513 | No difference | 0.0521 | No difference |

*Low or High or No difference lncRNA expression associated with worse survival.
