# Supplementary material for: In silico analyses identify lncRNAs: WDFY3-AS2, BDNF-AS and AFAP1-AS1 as potential prognostic factors for patients with triple-negative breast tumors
Source: PLoS One. 2020 May 13;15(5):e0232284. doi: 10.1371/journal.pone.0232284 (PMC7219740; doi:10.1371/journal.pone.0232284)
Supplement: S3 Table — (DOCX) [file pone.0232284.s009.docx]

**Suppl. Table 3** - Statistical analysis comparing lncRNA expression differences between normal tissue (104 cases) and tumor tissue (828 cases).

| **lncRNA** | **Mann-Whitney U test** | **Wilcoxon W** | **Z** | **Significance (bilateral)** |
| --- | --- | --- | --- | --- |
| WDFY3-AS2 | 3761,000 | 346967,000 | -15,186 | 0,000 |
| PRDM16-DT | 3674,000 | 324074,000 | -15,141 | 0,000 |
| LINC00548 | 10962,000 | 243865,000 | -11,166 | 0,000 |
| BDNF-AS | 21122,000 | 364328,000 | -8,477 | 0,000 |
| MNX1-AS1 | 17604,000 | 21975,000 | -7,638 | 0,000 |
| MIAT | 23394,000 | 28854,000 | -7,599 | 0,000 |
| LINC00339 | 24439,000 | 367645,000 | -7,195 | 0,000 |
| LINC02384 | 24857,000 | 362288,000 | -6,825 | 0,000 |
| ZNF205-AS1 | 26232,000 | 31692,000 | -6,502 | 0,000 |
| CNNM3-DT | 26964,000 | 367689,000 | -6,180 | 0,000 |
| LINC01018 | 29595,000 | 346801,000 | -4,732 | 0,000 |
| LINC00605 | 26572,000 | 31522,000 | -4,602 | 0,000 |
| LINC00205 | 32736,000 | 38196,000 | -3,988 | 0,000 |
| LINC00909 | 33038,000 | 376244,000 | -3,872 | 0,000 |
| LINC00494 | 29257,000 | 33628,000 | -3,035 | 0,002 |
| ATE1-AS1 | 35460,000 | 357463,000 | -2,487 | 0,013 |
| AFAP1-AS1 | 6197,000 | 7278,000 | -2,017 | 0,044 |
| MCF2L-AS1 | 37950,000 | 43410,000 | -1,973 | 0,048 |
| TGFB2-AS1 | 20827,000 | 153697,000 | -1,535 | 0,125 |
| LINC00618 | 31162,000 | 287132,000 | -1,147 | 0,251 |
| KDM7A-DT | 40472,000 | 383678,000 | -0,999 | 0,318 |
| PAXIP1-AS1 | 40818,000 | 384024,000 | -0,865 | 0,387 |
| HAGLR | 41307,000 | 384513,000 | -0,676 | 0,499 |

* Significance, significant according to p-value less than 0.05.
