# Supplementary material for: In silico analyses identify lncRNAs: WDFY3-AS2, BDNF-AS and AFAP1-AS1 as potential prognostic factors for patients with triple-negative breast tumors
Source: PLoS One. 2020 May 13;15(5):e0232284. doi: 10.1371/journal.pone.0232284 (PMC7219740; doi:10.1371/journal.pone.0232284)
Supplement: S4 Table — (DOCX) [file pone.0232284.s010.docx]

**Suppl. Table 4** - Statistical analysis comparing differences in lncRNA expression between intrinsic subtypes of the PAM50 classification.

| **lncRNA** | **Kruskal-Wallis H test** | **gl** | **Significance** |
| --- | --- | --- | --- |
| ZNF205-AS1 | 297,8616288 | 4 | 3,13372E-63 |
| WDFY3-AS2 | 193,5796882 | 4 | 9,01568E-41 |
| BDNF-AS | 125,0667915 | 4 | 4,41665E-26 |
| PRDM16-DT | 108,4159409 | 4 | 1,58409E-22 |
| AFAP1-AS1 | 100,7063152 | 4 | 6,95775E-21 |
| HAGLR | 89,89940631 | 4 | 1,38317E-18 |
| KDM7A-DT | 89,45937237 | 4 | 1,71531E-18 |
| MNX1-AS1 | 64,01433914 | 4 | 4,15022E-13 |
| TGFB2-AS1 | 53,23953208 | 4 | 7,59281E-11 |
| LINC00909 | 53,17806061 | 4 | 7,82109E-11 |
| LINC02384 | 33,45839464 | 4 | 9,62257E-07 |
| LINC01018 | 26,30351013 | 4 | 2,74837E-05 |
| LINC00339 | 24,82097183 | 4 | 5,46561E-05 |
| PAXIP1-AS1 | 22,7001399 | 4 | 0,000145344 |
| ATE1-AS1 | 22,2795021 | 4 | 0,00017631 |
| LINC00605 | 22,02261833 | 4 | 0,000198353 |
| LINC00205 | 19,84720546 | 4 | 0,000535303 |
| LINC00548 | 16,60665786 | 4 | 0,00230435 |
| CNNM3-DT | 15,53003033 | 4 | 0,003719199 |
| MCF2L-AS1 | 15,17448702 | 4 | 0,00435267 |
| LINC00618 | 11,2261842 | 4 | 0,024136242 |
| LINC00494 | 8,978595085 | 4 | 0,061636734 |
| MIAT | 8,062625713 | 4 | 0,089310885 |

* Significance, significant according to p-value less than 0.05.
