# Supplementary material for: In silico analyses identify lncRNAs: WDFY3-AS2, BDNF-AS and AFAP1-AS1 as potential prognostic factors for patients with triple-negative breast tumors
Source: PLoS One. 2020 May 13;15(5):e0232284. doi: 10.1371/journal.pone.0232284 (PMC7219740; doi:10.1371/journal.pone.0232284)
Supplement: S5 Table — (DOCX) [file pone.0232284.s011.docx]

**Suppl. Table 5** - Multiple analysis comparing lncRNAs expression in different breast cancer subtypes.

| **lncRNA** | **Mann-Whitney U test** | **Wilcoxon W** | **Z** | **Valor p** | **Sig/Not sig*** |
| --- | --- | --- | --- | --- | --- |
| **Basal vs HER2+** | | | | | |
| HAGLR | 1845 | 11575 | -7,014532579 | 0,000 | Significant |
| ZNF205-AS1 | 2156 | 11886 | -6,238605269 | 0,000 | Significant |
| PRDM16-DT | 2482 | 10357 | -4,394978499 | 0,000 | Significant |
| WDFY3-AS2 | 2901 | 12631 | -4,379872646 | 0,000 | Significant |
| LINC00339 | 3195 | 5473 | -3,646359369 | 0,000 | Significant |
| PAXIP1-AS1 | 3244 | 5522 | -3,524107156 | 0,000 | Significant |
| LINC00909 | 3277 | 5555 | -3,441774033 | 0,001 | Significant |
| CNNM3-DT | 3276 | 5487 | -3,303545834 | 0,001 | Significant |
| LINC00605 | 2386 | 4156 | -3,26993443 | 0,001 | Significant |
| AFAP1-AS1 | 553 | 743 | -3,269722581 | 0,001 | Significant |
| KDM7A-DT | 3526 | 13256 | -2,820533196 | 0,005 | Significant |
| MNX1-AS1 | 3209 | 11594 | -2,810328237 | 0,005 | Significant |
| BDNF-AS | 3531 | 5809 | -2,808058481 | 0,005 | Significant |
| MCF2L-AS1 | 3616 | 5894 | -2,595988315 | 0,009 | Not significant |
| LINC01018 | 3332 | 5412 | -2,535009498 | 0,011 | Not significant |
| TGFB2-AS1 | 1784 | 2604 | -2,488098392 | 0,013 | Not significant |
| LINC00205 | 3722 | 6000 | -2,331524345 | 0,020 | Not significant |
| ATE1-AS1 | 3454 | 5599 | -2,14907249 | 0,032 | Not significant |
| MIAT | 3852 | 13582 | -2,007181739 | 0,045 | Not significant |
| LINC00494 | 3414 | 11164 | -1,878541626 | 0,060 | Not significant |
| LINC00618 | 2721 | 4206 | -1,294647648 | 0,195 | Not significant |
| LINC00548 | 2804 | 9359 | -1,286405399 | 0,198 | Not significant |
| LINC02384 | 4399 | 13990 | -0,562248217 | 0,574 | Not significant |
| **Basal vs Luminal A** | | | | | |
| ZNF205-AS1 | 3340,000 | 13070,000 | -15,594 | 0,000 | Significant |
| WDFY3-AS2 | 7998,000 | 17728,000 | -12,730 | 0,000 | Significant |
| AFAP1-AS1 | 2205,000 | 10983,000 | -9,322 | 0,000 | Significant |
| BDNF-AS | 14983,000 | 24713,000 | -8,436 | 0,000 | Significant |
| HAGLR | 15257,000 | 24987,000 | -8,267 | 0,000 | Significant |
| PRDM16-DT | 15037,000 | 22912,000 | -6,919 | 0,000 | Significant |
| KDM7A-DT | 18023,000 | 103514,000 | -6,567 | 0,000 | Significant |
| TGFB2-AS1 | 8729,000 | 39110,000 | -6,441 | 0,000 | Significant |
| LINC00605 | 16616,000 | 93252,000 | -4,375 | 0,000 | Significant |
| LINC01018 | 20151,000 | 99154,000 | -4,199 | 0,000 | Significant |
| LINC02384 | 21540,000 | 105385,000 | -4,161 | 0,000 | Significant |
| MNX1-AS1 | 18276,000 | 85071,000 | -3,779 | 0,000 | Significant |
| LINC00205 | 22697,000 | 108188,000 | -3,693 | 0,000 | Significant |
| LINC00909 | 23370,000 | 33100,000 | -3,279 | 0,001 | Significant |
| MCF2L-AS1 | 24274,000 | 109765,000 | -2,723 | 0,006 | Not significant |
| LINC00494 | 21109,000 | 28859,000 | -2,246 | 0,025 | Not significant |
| MIAT | 25481,000 | 35211,000 | -1,981 | 0,048 | Not significant |
| LINC00548 | 17599,000 | 24154,000 | -1,683 | 0,092 | Not significant |
| CNNM3-DT | 26351,000 | 111017,000 | -1,367 | 0,172 | Not significant |
| LINC00618 | 19740,000 | 26410,000 | -0,749 | 0,454 | Not significant |
| ATE1-AS1 | 25525,000 | 105725,000 | -0,443 | 0,658 | Not significant |
| LINC00339 | 28141,000 | 37871,000 | -0,346 | 0,729 | Not significant |
| PAXIP1-AS1 | 28549,000 | 38279,000 | -0,095 | 0,924 | Not significant |
| **Basal vs Luminal B** | | | | | |
| ZNF205-AS1 | 1728,000 | 11458,000 | -13,363 | 0,000 | Significant |
| WDFY3-AS2 | 5704,000 | 15434,000 | -8,619 | 0,000 | Significant |
| HAGLR | 6519,000 | 16249,000 | -7,646 | 0,000 | Significant |
| AFAP1-AS1 | 1150,000 | 3103,000 | -7,207 | 0,000 | Significant |
| TGFB2-AS1 | 3021,000 | 7026,000 | -5,431 | 0,000 | Significant |
| KDM7A-DT | 8992,000 | 26383,000 | -4,695 | 0,000 | Significant |
| LINC01018 | 8433,000 | 24364,000 | -4,428 | 0,000 | Significant |
| LINC00205 | 9580,000 | 26971,000 | -3,994 | 0,000 | Significant |
| LINC00605 | 7059,000 | 20100,000 | -3,465 | 0,001 | Significant |
| MCF2L-AS1 | 10177,000 | 27568,000 | -3,281 | 0,001 | Significant |
| LINC02384 | 10243,000 | 27448,000 | -3,038 | 0,002 | Significant |
| BDNF-AS | 10503,000 | 20233,000 | -2,892 | 0,004 | Significant |
| LINC00909 | 10570,000 | 20300,000 | -2,812 | 0,005 | Significant |
| ATE1-AS1 | 9758,000 | 26594,000 | -2,809 | 0,005 | Significant |
| LINC00494 | 9160,000 | 16910,000 | -2,220 | 0,026 | Not significant |
| LINC00618 | 8258,000 | 14928,000 | -1,998 | 0,046 | Not significant |
| LINC00339 | 11345,000 | 28736,000 | -1,888 | 0,059 | Not significant |
| PRDM16-DT | 10668,000 | 18543,000 | -0,847 | 0,397 | Not significant |
| MNX1-AS1 | 9533,000 | 17918,000 | -0,674 | 0,500 | Not significant |
| LINC00548 | 7969,000 | 18700,000 | -0,587 | 0,557 | Not significant |
| CNNM3-DT | 12483,000 | 29874,000 | -0,530 | 0,596 | Not significant |
| MIAT | 12504,000 | 22234,000 | -0,505 | 0,614 | No tsignificant |
| PAXIP1-AS1 | 12752,000 | 22482,000 | -0,209 | 0,835 | Not significant |
| **Basal vs Normal-like** | | | | | |
| WDFY3-AS2 | 423,000 | 10153,000 | -5,641 | 0,000 | Significant |
| PRDM16-DT | 462,000 | 8337,000 | -4,958 | 0,000 | Significant |
| ZNF205-AS1 | 794,000 | 10524,000 | -3,861 | 0,000 | Significant |
| AFAP1-AS1 | 85,000 | 113,000 | -3,448 | 0,001 | Significant |
| HAGLR | 915,000 | 10645,000 | -3,280 | 0,001 | Significant |
| BDNF-AS | 1025,000 | 10755,000 | -2,752 | 0,0049 | Significant |
| LINC00548 | 768,000 | 7323,000 | -2,604 | 0,009 | Not significant |
| LINC00205 | 1080,000 | 1356,000 | -2,488 | 0,013 | Not significant |
| MNX1-AS1 | 899,000 | 1130,000 | -2,467 | 0,014 | Not significant |
| LINC00494 | 939,000 | 8689,000 | -2,325 | 0,020 | Not significant |
| MCF2L-AS1 | 1248,000 | 1524,000 | -1,682 | 0,093 | Not significant |
| PAXIP1-AS1 | 1253,000 | 1529,000 | -1,658 | 0,097 | No tsignificant |
| LINC00605 | 1070,000 | 1346,000 | -1,496 | 0,135 | Not significant |
| TGFB2-AS1 | 916,000 | 1106,000 | -1,421 | 0,155 | Not significant |
| ATE1-AS1 | 1306,000 | 9952,000 | -1,016 | 0,309 | Not significant |
| LINC00909 | 1389,000 | 11119,000 | -1,005 | 0,315 | Not significant |
| LINC00618 | 964,000 | 1154,000 | -0,820 | 0,412 | Not significant |
| LINC00339 | 1429,000 | 11159,000 | -0,813 | 0,416 | Not significant |
| KDM7A-DT | 1511,000 | 1787,000 | -0,420 | 0,675 | Not significant |
| MIAT | 1522,000 | 11252,000 | -0,367 | 0,714 | Not significant |
| CNNM3-DT | 1546,000 | 11276,000 | -0,252 | 0,801 | Not significant |
| LINC02384 | 1491,000 | 1744,000 | -0,134 | 0,894 | Not significant |
| LINC01018 | 1535,000 | 1811,000 | -0,030 | 0,976 | Not significant |

* Sig, significant; Not sig, significant according to p-value less than 0.005.
