# Supplementary material for: In silico analyses identify lncRNAs: WDFY3-AS2, BDNF-AS and AFAP1-AS1 as potential prognostic factors for patients with triple-negative breast tumors
Source: PLoS One. 2020 May 13;15(5):e0232284. doi: 10.1371/journal.pone.0232284 (PMC7219740; doi:10.1371/journal.pone.0232284)
Supplement: S6 Table — (DOCX) [file pone.0232284.s012.docx]

**Suppl. Table 6** - Frequency of cases by lncRNA according to expression data available from TANRIC database.

| **Patient characteristics** | **WDFY3-AS2** | | *Total* | **BDNF-AS** | | *Total* | **AFAP1-AS1** | | *Total* |
| --- | --- | --- | --- | --- | --- | --- | --- | --- | --- |
|  | High | Low |  | High | Low |  | High | Low |  |
| **Age, mean ± SD** | 58.7 ± 13.1 (26-90) | 57.4 ± 13.1 (26-90) |  | 58.0 ± 13.2 (26-90) | 58.1 ± 13.1 (26-90) |  | 55.6 ± 12.5 (29-85) | 57.6 ± 13.2 (26-90) |  |
| **Age, n (%)** |  |  |  |  |  |  |  |  |  |
| <50 | 111 (13.4) | 114 (13.8) | 225 (27.2) | 127 (15.3) | 98 (11.8) | 225 (27.2) | 59 (17.9) | 47 (14.2) | 106 (32.1) |
| ≥50 | 303 (36.6) | 300 (36.2) | 603 (72.8) | 287 (34.7) | 316 (38.2) | 603 (72.8) | 106 (32.1) | 118 (35.8) | 224 (67.9) |
| **Status vital, n (%)** |  |  |  |  |  |  |  |  |  |
| Alive | 348 (42.0) | 355 (42.9) | 703 (84.9) | 346 (41.8) | 357 (43.1) | 703 (84.9) | 141 (42.7) | 140 (42.4) | 281 (85.2) |
| Dead | 66 (8.0) | 59 (7.1) | 125 (15.1) | 68 (8.2) | 57 (6.9) | 125 (15.1) | 24 (7.3) | 25 (7.6) | 49 (14.8) |
| **Tumor stage, n (%)** |  |  |  |  |  |  |  |  |  |
| Stage I/II | 307 (37.1) | 305 (36.8) | 612 (73.9) | 316 (38.2) | 296 (35.7) | 612 (73.9) | 129 (39.1) | 124 (37.6) | 253 (76.7) |
| Stage III/IV | 91 (11.0) | 101 (12.2) | 192 (23.2) | 86 (10.4) | 106 (12.8) | 192 (23.2) | 32 (9.7) | 32 (9.7) | 64 (19.4) |
| Stage X | 12 (1.4) | 5 (0.6) | 17 (2.1) | 7 (0.8) | 10 (1.2) | 17 (2.1) | 4 (1.2) | 4 (1.2) | 8 (2.4) |
| Not reported | 4 (0.5) | 3 (0.4) | 7 (0.8) | 5 (0.6) | 2 (0.2) | 7 (0.8) | 0 (0.0) | 5 (1.5) | 5 (1.5) |
| **Estrogen Receptor** |  |  |  |  |  |  |  |  |  |
| Positive | 335 (40.5) | 240 (29.0) | 575 (69.4) | 324 (39.1) | 251 (30.3) | 575 (69.4) | 70 (21.2) | 116 (35.2) | 186 (56.4) |
| Negative | 34 (4.1) | 140 (16.9) | 174 (21.0) | 48 (5.8) | 126 (15.2) | 174 (21.0) | 79 (23.9) | 29 (8.8) | 108 (32.7) |
| Not reported | 45 (5.4) | 34 (4.1) | 79 (9.5) | 42 (5.1) | 37 (4.5) | 79 (9.5) | 16 (4.8) | 20 (6.1) | 36 (10.9) |
| **Progesterone Receptor** |  |  |  |  |  |  |  |  |  |
| Positive | 289 (34.9) | 211 (25.5) | 500 (60.4) | 288 (34.8) | 212 (25.6) | 500 (60.4) | 58 (17.6) | 102 (30.9) | 160 (48.5) |
| Negative | 79 (9.5) | 167 (20.2) | 246 (29.7) | 82 (9.9) | 164 (19.8) | 246 (29.7) | 88 (26.7) | 43 (13.0) | 131 (39.7) |
| Not reported | 46 (5.6) | 36 (4.3) | 82 (9.9) | 44 (5.3) | 38 (4.6) | 82 (9.9) | 19 (5.8) | 20 (6.1) | 39 (11.8) |
| **HER2** |  |  |  |  |  |  |  |  |  |
| Positive | 42 (5.1) | 65 (7.9) | 107 (12.9) | 28 (3.4) | 79 (9.5) | 107 (12.9) | 12 (3.6) | 21 (6.4) | 33 (10.0) |
| Negative | 322 (38.9) | 305 (36.8) | 627 (75.7) | 336 (40.6) | 291 (35.1) | 627 (75.7) | 140 (42.4) | 117 (35.5) | 257 (77.9) |
| Not reported | 50 (6.0) | 44 (5.3) | 94 (11.4) | 50 (6.0) | 44 (5.3) | 94 (11.4) | 13 (3.9) | 27 (8.2) | 40 (12.1) |
| **PAM50 Classification** |  |  |  |  |  |  |  |  |  |
| Basal | 21 (2.5) | 118 (14.3) | 139 (16.8) | 40 (4.8) | 99 (12.0) | 139 (16.8) | 88 (26.7) | 22 (6.7) | 110 (33.3) |
| HER2+ | 18 (2.2) | 49 (5.9) | 67 (8.1) | 13 (1.6) | 54 (6.5) | 67 (8.1) | 8 (2.4) | 11 (3.3) | 19 (5.8) |
| Luminal A | 278 (33.6) | 135 (16.3) | 413 (49.9) | 265 (32.0) | 148 (17.9) | 413 (49.9) | 48 (14.5) | 84 (25.5) | 132 (40.0) |
| Luminal B | 82 (9.9) | 104 (12.6) | 186 (22.5) | 85 (10.3) | 101 (12.2) | 186 (22.5) | 20 (6.1) | 42 (12.7) | 62 (18.8) |
| Normal-like | 15 (1.8) | 8 (1.0) | 23 (2.8) | 11 (1.3) | 12 (1.4) | 23 (2.8) | 1 (0.3) | 6 (1.8) | 7 (2.1) |
| *Total* | 414 (50.0) | 414 (50.0) | 828 (100.0) | 414 (50.0) | 414 (50.0) | 828 (100.0) | 165 (50.0) | 165 (50.0) | 330 (100.0) |
