# Supplementary material for: In silico analyses identify lncRNAs: WDFY3-AS2, BDNF-AS and AFAP1-AS1 as potential prognostic factors for patients with triple-negative breast tumors
Source: PLoS One. 2020 May 13;15(5):e0232284. doi: 10.1371/journal.pone.0232284 (PMC7219740; doi:10.1371/journal.pone.0232284)
Supplement: S7 Table — (DOCX) [file pone.0232284.s013.docx]

**Suppl. Table 7 -** Clinicopathological data of breast cancer patients from TANRIC-TCGA bank.

| **Patient characteristics** | **WDFY3-AS2** | | **P-value** | **BDNF-AS** | | **P-value** | **AFAP1-AS1** | | **P-value** |
| --- | --- | --- | --- | --- | --- | --- | --- | --- | --- |
|  | High | Low |  | High | Low |  | High | Low |  |
| **Age, n (%)** |  |  |  |  |  |  |  |  |  |
| <50 | 111 (13.4) | 114 (13.8) | 0,81 | 127 (15.3) | 98 (11.8) | 0.02* | 59 (17.9) | 47 (14.2) | 0,16 |
| ≥50 | 303 (36.6) | 300 (36.2) |  | 287 (34.7) | 316 (38.2) |  | 106 (32.1) | 118 (35.8) |  |
| **Race, n (%)** |  |  |  |  |  |  |  |  |  |
| Asian | 24 (2.9) | 30 (3.6) | 0,36 | 28 (3.4) | 26 (3.1) | 0,39 | 11 (3.3) | 10 (3.0) | 0,27 |
| Black/African American | 27 (3.3) | 38 (4.6) |  | 26 (3.1) | 39 (4.7) |  | 18 (5.5) | 6 (1.8) |  |
| White | 321 (38.8) | 300 (36.2) |  | 318 (38.4) | 303 (36.6) |  | 122 (37.0) | 125 (37.9) |  |
| American indian/Alaska native | 0 (0.0) | 1 (0.1) |  | 0 (0.0) | 1 (0.1) |  | 0 (0.0) | 0 (0.0) |  |
| Not reported | 42 (5.1) | 45 (5.4) |  | 42 (5.1) | 45 (5.4) |  | 14 (4.2) | 24 (7.3) |  |
| **Status vital, n (%)** |  |  |  |  |  |  |  |  |  |
| Alive | 348 (42.0) | 355 (42.9) | 0,56 | 346 (41.8) | 357 (43.1) | 0,33 | 141 (42.7) | 140 (42.4) | 0,98 |
| Dead | 66 (8.0) | 59 (7.1) |  | 68 (8.2) | 57 (6.9) |  | 24 (7.3) | 25 (7.6) |  |
| **Tumor stage, n (%)** |  |  |  |  |  |  |  |  |  |
| Stage I/II | 307 (37.1) | 305 (36.8) | 0,20 | 316 (38.2) | 296 (35.7) | 0,25 | 129 (39.1) | 124 (37.6) | 0,07 |
| Stage III/IV | 91 (11.0) | 101 (12.2) |  | 86 (10.4) | 106 (12.8) |  | 32 (9.7) | 32 (9.7) |  |
| Stage X | 12 (1.4) | 5 (0.6) |  | 7 (0.8) | 10 (1.2) |  | 4 (1.2) | 4 (1.2) |  |
| Not reported | 4 (0.5) | 3 (0.4) |  | 5 (0.6) | 2 (0.2) |  | 0 (0.0) | 5 (1.5) |  |
| **Estrogen Receptor** |  |  |  |  |  |  |  |  |  |
| Positive | 335 (40.5) | 240 (29.0) | <0.000*** | 324 (39.1) | 251 (30.3) | <0.000*** | 70 (21.2) | 116 (35.2) | <0.000*** |
| Negative | 34 (4.1) | 140 (16.9) |  | 48 (5.8) | 126 (15.2) |  | 79 (23.9) | 29 (8.8) |  |
| Not reported | 45 (5.4) | 34 (4.1) |  | 42 (5.1) | 37 (4.5) |  | 16 (4.8) | 20 (6.1) |  |
| **Progesterone Receptor** |  |  |  |  |  |  |  |  |  |
| Positive | 289 (34.9) | 211 (25.5) | <0.000*** | 288 (34.8) | 212 (25.6) | <0.000*** | 58 (17.6) | 102 (30.9) | <0.000*** |
| Negative | 79 (9.5) | 167 (20.2) |  | 82 (9.9) | 164 (19.8) |  | 88 (26.7) | 43 (13.0) |  |
| Not reported | 46 (5.6) | 36 (4.3) |  | 44 (5.3) | 38 (4.6) |  | 19 (5.8) | 20 (6.1) |  |
| **HER2** |  |  |  |  |  |  |  |  |  |
| Positive | 42 (5.1) | 65 (7.9) | <0.000*** | 28 (3.4) | 79 (9.5) | <0.000*** | 12 (3.6) | 21 (6.4) | <0.000*** |
| Negative | 322 (38.9) | 305 (36.8) |  | 336 (40.6) | 291 (35.1) |  | 140 (42.4) | 117 (35.5) |  |
| Not reported | 50 (6.0) | 44 (5.3) |  | 50 (6.0) | 44 (5.3) |  | 13 (3.9) | 27 (8.2) |  |
| **PAM50 Classification** |  |  |  |  |  |  |  |  |  |
| Basal | 21 (2.5) | 118 (14.3) | <0.000*** | 40 (4.8) | 99 (12.0) | <0.000*** | 88 (26.7) | 22 (6.7) | <0.000*** |
| HER2+ | 18 (2.2) | 49 (5.9) |  | 13 (1.6) | 54 (6.5) |  | 8 (2.4) | 11 (3.3) |  |
| Luminal A | 278 (33.6) | 135 (16.3) |  | 265 (32.0) | 148 (17.9) |  | 48 (14.5) | 84 (25.5) |  |
| Luminal B | 82 (9.9) | 104 (12.6) |  | 85 (10.3) | 101 (12.2) |  | 20 (6.1) | 42 (12.7) |  |
| Normal-like | 15 (1.8) | 8 (1.0) |  | 11 (1.3) | 12 (1.4) |  | 1 (0.3) | 6 (1.8) |  |
| **Primary diagnoses, n (%)** |  |  |  |  |  |  |  |  |  |
| Adenoid cystic carcinoma | 1 (0.1) | 0 (0.0) | 0.002** | 1 (0.1) | 0 (0.0) | 0.04* | 0 (0.0) | 1 (0.3) | 0.025* |
| Apocrine adenocarcinoma | 0 (0.0) | 1 (0.1) |  | 0 (0.0) | 1 (0.1) |  | 0 (0.0) | 0 (0.0) |  |
| Basal cell carcinoma | 0 (0.0) | 1 (0.1) |  | 1 (0.1) | 0 (0.0) |  | 0 (0.0) | 0 (0.0) |  |
| Cribriform carcinoma | 0 (0.0) | 1 (0.1) |  | 1 (0.1) | 0 (0.0) |  | 0 (0.0) | 0 (0.0) |  |
| Infiltrating duct and lobular carcinoma | 18 (2.2) | 7 (0.8) |  | 13 (1.6) | 12 (1.4) |  | 4 (1.2) | 5 (1.5) |  |
| Infiltrating duct carcinoma | 304 (36.7) | 346 (41.8) |  | 308 (37.2) | 342 (41.3) |  | 129 (39.1 | 128 (38.8) |  |
| Infiltrating duct mixed with other types of carcinoma | 13 (1.6) | 5 (0.6) |  | 12 (1.4) | 6 (0.7) |  | 2 (0.6) | 0 (0.0) |  |
| Infiltrating lobular mixed with other types of carcinoma | 1 (0.1) | 1 (0.1) |  | 2 (0.2) | 0 (0.0) |  | 0 (0.0) | 0 (0.0) |  |
| Intraductal micropapillary carcinoma | 0 (0.0) | 3 (0.4) |  | 1 (0.1) | 2 (0.2) |  | 0 (0.0) | 1 (0.3) |  |
| Intraductal papillary adenocarcinoma with invasion | 3 (0.4) | 1 (0.1) |  | 3 (0.4) | 1 (0.1) |  | 1 (0.3) | 1 (0.3) |  |
| Large cell neuroendocrine carcinoma | 0 (0.0) | 1 (0.1) |  | 0 (0.0) | 1 (0.1) |  | 1 (0.3) | 0 (0.0) |  |
| Lobular carcinoma | 66 (8.0) | 32 (3.9) |  | 62 (7.5) | 36 (4.3) |  | 18 (5.5) | 28 (8.5) |  |
| Medullary carcinoma | 0 (0.0) | 4 (0.5) |  | 1 (0.1) | 3 (0.4) |  | 3 (0.9) | 0 (0.0) |  |
| Metaplastic carcinoma | 2 (0.2) | 4 (0.5) |  | 1 (0.1) | 5 (0.6) |  | 5 (1.5) | 0 (0.0) |  |
| Mucinous adenocarcinoma | 4 (0.5) | 4 (0.5) |  | 6 (0.7) | 2 (0.2) |  | 1 (0.3) | 1 (0.3) |  |
| Paget disease and infiltrating duct carcinoma of breast | 1 (0.1) | 1 (0.1) |  | 0 (0.0) | 2 (0.2) |  | 0 (0.0) | 0 (0.0) |  |
| Papillary carcinoma | 0 (0.0) | 1 (0.1) |  | 1 (0.1) | 0 (0.0) |  | 0 (0.0) | 0 (0.0) |  |
| Pleomorphic carcinoma | 0 (0.0) | 1 (0.1) |  | 0 (0.0) | 1 (0.1) |  | 0 (0.0) | 0 (0.0) |  |
| Secretory carcinoma of breast | 1 (0.1) | 0 (0.0) |  | 1 (0.1) | 0 (0.0) |  | 1 (0.3) | 0 (0.0) |  |

* P<0.05, ** p<0.01, *** p<0.001
